# Closing the ODE–SDE gap in score-based diffusion models through the Fokker–Planck equation

**DOI:** 10.1098/rsta.2024.0503

**Published:** 2025-06-05

**Authors:** Teo Deveney, Jan Stanczuk, Lisa Kreusser, Chris Budd, Carola-Bibiane Schönlieb

**Affiliations:** ^1^ Department of Computer Science, University of Bath, Bath, UK; ^2^ Department of Applied Mathematics and Theoretical Physics, University of Cambridge, Cambridge, Cambridgeshire, UK; ^3^ Department of Mathematical Sciences, University of Bath, Bath, UK

**Keywords:** score-based diffusion, Fokker–Planck, generative modelling, Wasserstein distance

## Abstract

Score-based diffusion models have emerged as one of the most promising frameworks for deep generative modelling, due to both their mathematical foundations and their state-of-the art performance in many tasks. Empirically, it has been reported that samplers based on ordinary differential equations (ODEs) are inferior to those based on stochastic differential equations (SDEs). In this article, we systematically analyse the difference between the ODE and SDE dynamics of score-based diffusion models and show how this relates to an associated Fokker–Planck equation. We rigorously describe the full range of dynamics and approximations arising when training score-based diffusion models and derive a theoretical upper bound on the Wasserstein 2-distance between the ODE- and SDE-induced distributions in terms of a Fokker–Planck residual. We also show numerically that conventional score-based diffusion models can exhibit significant differences between ODE- and SDE-induced distributions that we demonstrate using explicit comparisons. Moreover, we show numerically that reducing this Fokker–Planck residual by adding it as an additional regularization term during training closes the gap between ODE- and SDE-induced distributions. Our experiments suggest that this regularization can improve the distribution generated by the ODE; however this can come at the cost of degraded SDE sample quality.

This article is part of the theme issue ‘Partial differential equations in data science’.

## 1. Introduction

Generative modelling, the task of approximating the distribution underlying some given dataset, is useful in a range of scientific and non-scientific applications. The current state-of-the-art are diffusion models [1,2], which obtain this approximation by perturbing data with white noise and learning to iteratively denoise the perturbed data. Around the same time score-based models [3,4], which approximate the distribution through the gradient of its log-density (the score function), showed impressive results when combined with Langevin-based sampling. Today these frameworks have been unified into a single score-based diffusion approach [5], where stochastic differential equations (SDE) or ordinary differential equations (ODE) driven by score models are applied to denoise perturbed data. This has received a lot of attention from both theoretical and applied communities due its strong mathematical foundation and state-of-the-art performance [5,6]. In practice, differences between SDE and ODE-based sampling distributions are observed even for a common score model, thus motivating this work.

The SDE formulation arises from the conversion of data into noise through a diffusion process. As score-based diffusion is used for data generation, the time reversal of this process, i.e. the conversion from noise to data, is crucial and has a closed-form expression that depends on the (unknown) time-dependent score function of this diffusion. Hence, data generation is achieved by approximating these reverse dynamics through a neural approximation of the score function. The ODE framework, which originates from a diffusion-free reparameterization of the Fokker–Planck equation, offers a deterministic sampling method that offers significant theoretical and computational benefits such as tractable likelihood computation and access to more efficient ODE integrators for faster sample generation. However, the existing literature indicates that the ODE-based samplers are inferior to the SDE-based samplers in diffusion models. This is evidenced empirically in [5], reporting lower Fréchet inception distances (an image quality metric) for ODE-based samplers in their experiments. Moreover, theoretical analysis corroborates this observation, where tighter upper bounds have been derived for SDE-based sampling than for the ODE [7–11]. These discrepancies in performance raise questions about the validity of the likelihood computations attained in practice and prompt us to investigate the reasons for the discrepancy between SDE- and ODE-induced distributions.

In this article, we investigate the ODE–SDE gap in score-based diffusion models theoretically by analysing their connection to the mechanism underpinning their relationship—the Fokker–Planck equation. Our aim is to expose the theoretical insight that the ODE–SDE gap is related to how well the score model approximates the solution to a Fokker–Planck equation. As such, our work makes use of tools from the analysis of partial differential equations (PDEs), combined with methods for ODEs and SDEs. In addition, we provide numerical experiments using the Fokker–Planck equation to construct a regularizer. For toy examples in two dimensions, we attain explicit visual comparisons of the distributions generated by ODE and SDE samples and measure the relevant Wasserstein distances. We emphasize that the objective of our numerics is to accurately support our theory in an interpretable way. Since our approach to the numerics is more costly than a traditional diffusion model to train (though the cost of sampling from the trained model is unchanged), it is not proposed to be a scalable approach to training high-dimensional models, and we refer to [12] for more scalable approximate approaches in this direction.

### (a) Related work

The deterministic ODE dynamics for score-based diffusion models were introduced in [5]. To compare the ODE and SDE distributions, the authors in [5] show that under a perfect score approximation, the SDE and ODE distributions coincide and derive a method for computing the likelihoods based on the ODE formulation. In the same work, it is empirically reported that the ODE sampler exhibits inferior performance. This empirical finding highlights the necessity for a more rigorous theoretical investigation into this phenomenon. In [13], the authors bound the Kullback–Leibler divergence between the SDE-induced model distribution and the target true distribution in terms of the score-matching objective minimized during training. However, they also point out that the same bound does not hold for the ODE-induced distribution.

This issue is further explored in [14], where the authors introduce a new equality that can be used for bounds of the Kullback–Leibler divergence between the ODE-induced distribution and the data-generating distribution. Their findings reveal that the conventional score matching objective, typically employed in score-based diffusion models, fails to adequately control the error in the ODE distribution, and an alternative objective is proposed.

Since then, both the ODE and SDE formulation have been analysed. For SDE-based sampling, theoretical convergence guarantees including polynomial-in-time convergence under an $L^{2}$ score approximation have been proven [7–11]. For ODE-sampling, fast convergence (polynomial-in-time) has only been shown with the presence of Langevin-based correction steps [15]. Unfortunately, this approach results in a stochastic sampler that sacrifices deterministic mappings and is unsuitable for likelihood computations. The analysis of the fully deterministic system includes [16], which establishes an upper bound based on the number of steps of the discretized ODE. In addition, [17] provides error bounds for the flow matching method introduced in [18], a generalization of diffusion-based ODE methods. Of these works, the bound in [17] looks most similar to ours, though none are directly applicable to our setting, since they are all comparisons to the ground truth given in terms of an $L^{2}$ score approximation, whereas we investigate the ODE–SDE gap in terms of a Fokker–Planck residual.

More closely related to our work, the authors in [12] relate the Kullback–Leibler divergence between ODE-induced and data-generating distributions to the error in the Fokker–Planck equation associated with the diffusion process. They demonstrate that the residual of the Fokker–Planck equation can bound the ODE sample error up to some non-zero threshold. For full convergence, their analysis additionally requires minimization of the score-matching objective.

Our work shares some themes with [12], since we also consider the Fokker–Planck equation underlying the diffusion dynamics. However our focus here differs, as we specifically focus on the ODE–SDE gap. Moreover, our analysis has been conducted independently using a different theoretical toolbox, and this reveals different insights as highlighted in §1b.

### (b) Contributions

In this work, we provide a concise and rigorous exposition of the full range of densities and their approximations that arise in the score-based diffusion framework. This includes densities of the true and approximate dynamics—both deterministic and stochastic, as well as forward and backwards in time—with their density evolution equations and the neural approximations producing them. Given these dynamics, the contributions of this work are the following:

— We derive an upper bound between the densities induced by the approximate ODE and the approximate SDE. To the authors’ knowledge, this is the first such bound that does not relate each approximated density to the true one. The distance is key when querying both the ODE and the SDE of the same model, for example when stochastic sampling is combined with likelihood evaluations.
  — Our bound is in Wasserstein-distance, in contrast to previous works that mostly focus on weaker distances such as Kullback–Leibler divergence or total variation.
  — Our bound relates the distributions through the Fokker–Planck equation of the generative process. This distinguishes our work from prior works in the area and is the pertinent object to study since ODE-based sampling arises through a reformulation of the Fokker–Planck equation. We show that the ODE–SDE gap increases when the neural approximation fails to satisfy a Fokker–Planck equation.
  — We prove our result for both the potential parameterization, and the score parameterization more commonly used in practice, by considering the analogous Fokker–Planck equation for the score function.
— We support our theory by providing numerical experiments using the residual of the Fokker–Planck equation in a regularization term. We visualize the densities and calculate the relevant Wasserstein distances to explicitly demonstrate that a lower residual error in the Fokker–Planck equation is associated with a lower ODE–SDE gap.

### (c) Outline

In §2, we describe the broad range of dynamics and approximations that arise when training a score-based diffusion model. Our main theoretical result on the ODE–SDE gap in score-based diffusion models is proven in §3, where we derive an upper bound on the Wasserstein 2-distance between the ODE- and SDE-induced distributions in terms of a Fokker–Planck residual. In §4, we provide numerical evidence showing explicitly that conventional score-based diffusion models can exhibit significant differences between SDE- and ODE-induced distributions. Moreover, we show that reducing the Fokker–Planck residual by adding it as an additional regularization term indeed leads to closing the gap between SDE and ODE distributions.

## 2. Score-based diffusion models

### (a) Assumptions and notation

We will work in the time domain t∈[0,T] for some T>0 and spatial domain $x\in \Omega \subset {\mathbb{R}}^{d}$ . For two vectors $a,b\in {\mathbb{R}}^{d}$ , we denote their inner product $a^{T}b$ , with associated norm $\|a{\|}_{2}$ . For a function $h:\Omega \to {\mathbb{R}}^{n}$ , we denote the $L^{2}$ -norm over some domain $\Omega \subset {\mathbb{R}}^{d}$ as $\|h{\|}_{L^{2}(\Omega )}$ . We denote by ∇ the (spatial) gradient and by $\nabla^{2}$ the Laplacian. For two probability measures, p,q on Ω , we denote their Wasserstein 2-distance by $W_{2}(p,q)$ .

Let (Ω,F,P) be a probability space and let $F_{t}\subset F$ be the natural filtration (the increasing family of sub- σ -algebras containing information at times [0,t] ). As is convention, we denote by $W_{t}\in {\mathbb{R}}^{d}$ a Brownian motion at time t with values in ${\mathbb{R}}^{d}$ adapted to the filtration $F_{t}$ . Conversely, let ${\bar{F}}_{t}\subset F$ denote a reverse filtration (the decreasing family of sub- σ -algebras containing information at times [t,T] ). We denote by ${\bar{W}}_{t}\in {\mathbb{R}}^{d}$ a Brownian motion at time t with values in ${\mathbb{R}}^{d}$ adapted to ${\bar{F}}_{t}$ . Under suitable assumptions, SDEs driven by $W_{t}$ are adapted to $F_{t}$ , and SDEs driven by ${\bar{W}}_{t}$ are adapted to ${\bar{F}}_{t}$ . Throughout we will refer to the former as *forward* SDEs, and the latter as *reverse* SDEs even though both SDEs will initially be formulated using the forward time variable t . When dealing with SDEs and the associated Fokker–Planck equations, we will distinguish between evolution equations running forward and backwards in time by introducing the reverse time variable τ=T−t to specify that the corresponding dynamics are in reverse time. We will denote the SDE dynamics parameterized with the forward and reverse time variables t and τ=T−t by $x_{t}$ and ${\bar{x}}_{\tau }$ , respectively, with ${\bar{x}}_{\tau }={\bar{x}}_{T-t}=x_{t}=x_{T-\tau }$ . For any function $h:{\mathbb{R}}^{d}\times [0,T]\to \mathbb{R}$ *,* we introduce $\bar{h}:{\mathbb{R}}^{d}\times [0,T]\to \mathbb{R}$ by $\bar{h}(\cdot ,\tau )=h(\cdot ,T-\tau )$ for all τ∈[0,T] . Further, let probability densities $p_{0},\pi$ on ${\mathbb{R}}^{d}$ be given, and we denote the associated log-densities by $u_{0}=\log\;p_{0}$ , $u_{T}=\log\;\pi$ on ${\mathbb{R}}^{d}$ . Throughout the article, we make the following regularity assumptions:

**Assumptions 2.1.** *Let* T>0 *and let* $f\in C^{\infty }({\mathbb{R}}^{d}\times [0,T];{\mathbb{R}}^{d})$ *such that* $\|f(x,t){\|}_{2}\leq K_{f}(1+\|x{\|}_{2})$ *for some* $K_{f}>0$ . *Assume that* $g\in C^{\infty }([0,T];\mathbb{R})$ *and there is* 0<m<M<∞ *such that* m≤g(t)≤M *for all* t∈[0,T] . *We assume that* $\Omega \subset {\mathbb{R}}^{d}$ *is a bounded domain with* $\partial \Omega \in C^{\infty }$ . *For neural approximations, we assume smooth activation functions, so that neural potential models* $u_{\theta }:{\mathbb{R}}^{d}\times [0,T]\to \mathbb{R}$ *are in* $C^{\infty }({\mathbb{R}}^{d}\times [0,T];\mathbb{R})$ *throughout, and neural score models* $s_{\theta }:{\mathbb{R}}^{d}\times [0,T]\to {\mathbb{R}}^{d}$ *are in* $C^{\infty }({\mathbb{R}}^{d}\times [0,T];{\mathbb{R}}^{d})$ . *Moreover, we assume that there are* $K_{u},K_{s}>0$ *such that* $\|\nabla u_{\theta }(x,t){\|}_{2}\leq K_{u}(1+\|x{\|}_{2})$ *and* $\|s_{\theta }(x,t){\|}_{2}\leq K_{s}(1+\|x{\|}_{2})$ . *Finally, we assume that the second moments of* $p_{0}$ *and* π *are finite, and that* $\pi \in C^{\infty }({\mathbb{R}}^{d};\mathbb{R})$ .

### (b) Particle dynamics

We introduce the *forward SDE* as


(2.1) $$\begin{array}{r} \text{d}x_{t}=f(x_{t},t)\text{d}t+g(t)dW_{t}, \end{array}$$

equipped with some initial distribution $p_{0}$ for $x_{0}$ . In generative modelling settings, this initial distribution $p_{0}$ represents the underlying target distribution from which the data were sampled. In equation (2.1), $W_{t}$ denotes the value of a Brownian motion adapted to $F_{t}$ , and therefore $x_{t}$ is also adapted to $F_{t}$ . We denote the associated marginal density of samples from equation (2.1) at time t by p(⋅,t) with $p_{0}=p(\cdot ,0)$ . Note that equation (2.1) has a unique t -continuous solution by assumptions 2.1.

In [19], the author shows that the process in equation (2.1) can be written as an SDE measurable with respect to the reverse filtration ${\bar{F}}_{t}$ . We refer to this SDE as the *reverse SDE*, and it is given by


(2.2) $$\begin{array}{r} \text{d}x_{t}=(f(x_{t},t)-g^{2}(t)\nabla \log p(x_{t},t))dt+g(t)d{\bar{W}}_{t}, \end{array}$$

where ${\bar{W}}_{t}$ is a Brownian motion adapted to ${\bar{F}}_{t}$ at time t . Intuitively, one can think of ${\bar{W}}_{t}$ as the backwards evolution of Brownian motion with known terminal state, and equation (2.2) as the backwards evolution of equation (2.1). Accordingly, if the terminal distribution for $x_{T}$ is set to p(⋅,T) *,* then the trajectories of equation (2.2) share the same distribution as equation (2.1) for any time t∈[0,T] . As shown in [5], a reformulation of the Fokker–Planck equations allows us to derive the *probability flow ODE* of the forward SDE equation (2.1). This is given by


(2.3) $$\begin{array}{r} \frac{\text{d}x_{t}}{\text{d}t}=f(x_{t},t)-\frac{1}{2}g^{2}(t)\nabla \log p(x_{t},t), \end{array}$$

equipped with initial distribution $p_{0}$ for $x_{0}$ or, equivalently, terminal distribution p(⋅,T) for $x_{T}$ . The trajectories initialized from $p_{0}$ evolve forward in time according to equation (2.3) and also have marginal distribution p(⋅,t) at time t . Similarly, the trajectories with terminal condition $x_{T}$ sampled from p(⋅,T) have marginal distribution p(⋅,t) at time t . Therefore, we have that the associated densities toequations equations (2.1), (2.2) and (2.3) are all given by p(⋅,t) at any time t∈[0,T] .

### (c) Neural approximation

For generative tasks, practitioners assume p(⋅,T) to be equal to a given *prior distribution* π and simulate equation (2.2) or (2.3) to generate samples from $p_{0}$ . Typically, π approximates p(⋅,T) and is an easy to sample from distribution that contains no information of $p_{0}$ , such as a Gaussian distribution with fixed mean and variance. However, solving equation (2.2) or (2.3) requires knowledge of the (*Stein) score function* $\nabla \log\;p(x_{t},t)\in {\mathbb{R}}^{d}$ for any $x_{t}$ , which is not known in general and must be approximated from data. Therefore, a neural network $s_{\theta }(x_{t},t)\in {\mathbb{R}}^{d}$ with model parameters θ is trained to approximate the score function from the data by minimizing the weighted score matching objective:


(2.4) $$\begin{array}{r} L_{SM}\left(\theta ,s_{\theta },\lambda \right):=E_{\begin{array}{c} t∼U(0,T)\\ x_{t}∼p\left(x_{t},t\right) \end{array}}\left[\lambda (t){\left\|\nabla \log p\left(x_{t},t\right)-s_{\theta }\left(x_{t},t\right)\right\|}_{2}^{2}\right], \end{array}$$

where $\lambda :[0,T]\;\overset{\;}{\to }\;{\mathbb{R}}_{+}$ is a positive weighting function.

$L_{SM}$ in equation (2.4) cannot be minimized directly since we do not have access to the ground truth score $\nabla \log\;p(x_{t},t)$ . Therefore, in practice, a different objective has to be used [3,5,20]. In [5], the weighted denoising score-matching objective is considered, which is defined as


(2.5) $$\begin{array}{r} L_{DSM}\left(\theta ,s_{\theta },\lambda \right):=E_{\begin{array}{c} t∼U(0,T)\\ x_{0}∼p_{0}(x_{0})\\ x_{t}∼p(x_{t},t|x_{0},0) \end{array}}\;\left[\lambda (t){\left\|\nabla \log p\left(x_{t},t|x_{0},0\right)-s_{\theta }\left(x_{t},t\right)\right\|}_{2}^{2}\right]. \end{array}$$

The difference between equations equations (2.4) and (2.5) is the replacement of the unknown ground truth score $\nabla \log\;p(x_{t},t)$ by the score of the perturbation kernel $\nabla \log\;p(x_{t},t|x_{0},0)$ , which can be determined analytically for many choices of forward SDEs. Note that for a fixed function λ , objective (2.5) is equal to objective (2.4) up to an additive constant, which does not depend on the model parameters θ . The reader can refer to [20] for the proof.

The choice of the weighting function λ determines the importance of score-matching at different noise scales. A principled choice is $\lambda (t)=g(t{)}^{2}$ , known as the likelihood weighting due to its relation to likelihood-based training (see discussion in appendix E).

Most implementations of neural score approximations parameterize the time-dependent score vector field directly with a neural network $s_{\theta }:\Omega \times [0,T]\;\overset{\;}{\to }\;{\mathbb{R}}^{d}$ on some bounded domain Ω . Such approximations generally result in a non-conservative vector, which therefore cannot be a gradient field of any scalar field (see, for example, figure 5 in appendix D). Since we know *a priori* that the target vector field ∇log⁡p is a gradient field, instead of learning $s_{\theta }$ , we consider a neural network $\phi_{\theta }\in C^{\infty }(\Omega \times [0,T];\mathbb{R})$ such that $\phi_{\theta }(x,t)$ approximates the log-density log⁡p(x,t) for any (x,t)∈Ω×[0,T] up to some normalizing constant. In other words, there exists a (time-dependent) normalizing constant $Z_{t}\in \mathbb{R}$ such that


$$p_{\theta }(x,t)=\exp(\phi_{\theta }(x,t)-\log\;Z_{t})$$

is a probability distribution. We write $u_{\theta }=\log\;p_{\theta }$ for the induced log-density, and we call the function $\phi_{\theta }(x,t)$ a *potential model*. During training, the induced approximate score $\nabla \phi_{\theta }(x,t)=\nabla u_{\theta }(x,t)\approx \nabla \log\;p(x,t)$ is computed by back-propagation through $\phi_{\theta }$ with respect to the input x . This results in a score approximation that is provably a conservative vector field. Moreover, it enables us to calculate the time derivative of the approximate log-density (up to normalization) as $\partial_{t}u_{\theta }(x,t)\approx \partial_{t}\log\;p(x,t)$ by back-propagation through $\phi_{\theta }$ with respect to t , which will be convenient when we introduce and evaluate a log-Fokker–Planck residual for $u_{\theta }$ in §2f.

### (d) Approximate particle dynamics

The above neural approximations $u_{\theta }=\log\;p_{\theta }\in C^{\infty }(\Omega \times [0,T];\mathbb{R})$ induce approximate versions of equation (2.2) and its deterministic flow equation (2.3). For ease of notation, we introduce the approximated reverse drift:


(2.6) $$\begin{array}{r} f_{\theta }^{\text{SDE}}(x,t)=f(x,t)-g^{2}(t)\nabla u_{\theta }(x,t), \end{array}$$

obtained by substituting the potential model into the drift of equation (2.2). Note that by the assumed properties of $f,g,u_{\theta }$ in assumptions 2.1, it follows that $f_{\theta }^{\text{SDE}}\in C^{\infty }(R^{d}\times [0,T];R^{d})$ and $\left\|f_{\theta }^{\text{SDE}}(x,t){\|}_{2}\leq (K_{f}+M^{2}K_{u})(1+\|x{\|}_{2}\right)$ . Using the approximated reverse drift equation (2.6) , we obtain the *reverse approximate SDE*:


(2.7) $$\begin{array}{r} \text{d}x_{t}=f_{\theta }^{\text{SDE}}(x_{t},t)\text{d}t+g(t)d{\bar{W}}_{t}, \end{array}$$

which can be regarded as an approximation of equation (2.2). Here, $x_{t}$ is adapted to the reverse time filtration ${\bar{F}}_{t}$ and by assumptions 2.1, equation (2.7) has a unique t -continuous solution. We denote the marginal density of $x_{t}$ satisfying equation (2.7) by $p_{\theta }^{\text{SDE}}(\cdot ,t)$ at time t and equip it with some terminal distribution π of $x_{T}$ at time T , i.e. $p_{\theta }^{\text{SDE}}(\cdot ,T)=\pi$ *,* where π is chosen to be a Gaussian approximation of p(⋅,T) . Thus the accuracy of the reverse flow of probability $p_{\theta }^{\text{SDE}}$ induced by equation (2.7) depends on the accuracy of the potential model. Applying the result of [19] to write equation (2.7) as a process measurable with respect to $F_{t}$ , we arrive at the *forward approximate SDE*, given by


(2.8) $$\begin{array}{r} dx_{t}=\left[f_{\theta }^{\text{SDE}}(x_{t},t)+g^{2}(t)\nabla \log p_{\theta }^{\text{SDE}}(x_{t},t)\right]dt+g(t)dW_{t}, \end{array}$$

where $x_{0}$ is drawn from $p_{\theta }^{\text{SDE}}(\cdot ,0)$ . The associated probability flow ODE of the approximate SDE (in forward time) is


(2.9) $$\begin{array}{r} \frac{dx_{t}}{dt}=f_{\theta }^{\text{SDE}}(x_{t},t)+\frac{1}{2}g^{2}(t)\nabla \log p_{\theta }^{\text{SDE}}(x_{t},t), \end{array}$$

where $x_{0}$ is drawn from $p_{\theta }^{\text{SDE}}(\cdot ,0)$ . Note that the associated densities to equations (2.7), (2.8) and (2.9) are all given by $p_{\theta }^{\text{SDE}}(\cdot ,t)$ for t∈[0,T] .

Finally, an approximation of the probability flow ODE equation (2.3) arises by approximating log⁡p in equation (2.3) by a neural network $u_{\theta }$ . This yields the *approximate probability flow ODE* (in forward time):


(2.10) $$\begin{array}{r} \frac{\text{d}x_{t}}{\text{d}t}=f_{\theta }^{\;\;ODE}(x_{t},t), \end{array}$$

using the approximate forward drift


(2.11) $$\begin{array}{r} f_{\theta }^{\;\;ODE}(x_{t},t)=f(x_{t},t)-\frac{1}{2}g^{2}(t)\nabla u_{\theta }(x_{t},t)\in C^{\infty }(R^{d}\times [0,T];R^{d}). \end{array}$$

Here, $x_{T}$ is distributed according to π . We denote the associated density $p_{\theta }^{ODE}(\cdot ,t)$ for t∈[0,T] .

In summary, the original formulations equations (2.1), (2.2) and (2.3) all have density p , the approximations equations (2.7), (2.8) and (2.9) obtained by approximating the reverse SDE equation (2.2) all have density $p_{\theta }^{\text{SDE}}$ and the approximation of the probability flow ODE equation (2.3) has density $p_{\theta }^{ODE}$ . Moreover, there is a density $p_{\theta }=\exp(u_{\theta })$ implied directly by the neural approximation to log-density. In general, we have that $p\neq p_{\theta }\neq p_{\theta }^{\text{SDE}}\neq p_{\theta }^{ODE}$ .

For the majority of our calculations and numerics, it is more convenient to work with logarithms of densities rather than the densities themselves. For each density p , we denote the associated log-density by u and refer to u as log-density or potential. That is, u(x,t)=log⁡p(x,t) *,* $u_{\theta }^{\text{SDE}}(x,t)=\log p_{\theta }^{\text{SDE}}(x,t)$ *,* $u_{\theta }^{ODE}(x,t)=\log\;p_{\theta }^{ODE}(x,t)$ and $u_{\theta }(x,t)=\log\;p_{\theta }(x,t)$ for all (x,t)∈Ω×[0,T] .

In addition to considering the dynamics in forward time, we can also introduce the dynamics in reverse time. We denote the reverse time dynamics by ${\bar{x}}_{\tau }$ for τ∈[0,T] satisfying ${\bar{x}}_{\tau }=x_{T-\tau }$ which implies that ${\bar{x}}_{T}=x_{0}$ and ${\bar{x}}_{0}=x_{T}$ for the initial and terminal conditions, respectively.

As we have a terminal condition $x_{T}$ for equation (2.7) and equation (2.7) is stated in forward time, the corresponding parameterization in reverse time can be useful for obtaining samples satisfying equation (2.7). It is given by


(2.12) $$\begin{array}{r} d{\bar{x}}_{\tau }=-{\bar{f}}_{\theta }^{\text{SDE}}({\bar{x}}_{\tau },\tau )d\tau -\bar{g}(\tau )dW_{\tau }, \end{array}$$

where we use the notation from §2a and the reverse time variable τ=T−t . We equip equation (2.12) with initial condition ${\bar{x}}_{0}$ which is sampled from π , and we denote the distribution of ${\bar{x}}_{\tau }$ at time τ by ${\bar{p}}_{\theta }^{\text{SDE}}(\cdot ,\tau )$ . Note that ${\bar{p}}_{\theta }^{\text{SDE}}(\cdot ,\tau )=p_{\theta }^{\text{SDE}}(\cdot ,T-\tau )$ for τ∈[0,T] with ${\bar{p}}_{\theta }^{\text{SDE}}(\cdot ,0)=\pi$ . This implies that we can sample a particle from the target distribution $p_{\theta }^{\text{SDE}}(\cdot ,0)$ by sampling ${\bar{x}}_{0}$ from π and solving equation (2.12) until time τ=T , for instance with the Euler–Maruyama scheme.

Similarly the ODE dynamics equation (2.10) can be written using the reverse time variable τ as


(2.13) $$\begin{array}{r} \frac{\text{d}{\bar{x}}_{\tau }}{\text{d}\tau }=-{\bar{f}}_{\theta }^{\;\;ODE}({\bar{x}}_{\tau },\tau ). \end{array}$$

To sample from the approximate target distribution $p_{\theta }^{ODE}(\cdot ,0)={\bar{p}}_{\theta }^{ODE}(\cdot ,T)$ *,* sample an initial condition ${\bar{x}}_{0}$ from ${\bar{p}}_{\theta }^{ODE}(\cdot ,0)=\pi$ and simulate equation (2.13) forward in time.

### (e) Fokker–Planck equations

The evolution of the densities subject to some initial or terminal condition are described by Fokker–Planck equations. For the forward SDE equation (2.1) the density p obeys the *forwardFokker–Planck equation*:


(2.14) $$\begin{array}{r} \frac{\partial p}{\partial t}(x,t)+\nabla \cdot (f(x,t)p(x,t))-\frac{1}{2}g^{2}(t)\nabla^{2}p(x,t)=0 \end{array}$$

on ${\mathbb{R}}^{d}\times \left[0,T\right]$ , equipped with the initial data $p_{0}$ on the full space ${\mathbb{R}}^{d}$ . For our analysis, we restrict ourselves to a bounded domain $\Omega \subset {\mathbb{R}}^{d}$ with $\partial \Omega \in C^{\infty }$ . For considering equation (2.14) on Ω , we equip equation (2.14) with positive Dirichlet boundary conditions. Let $p_{B}:\partial \Omega \times [0,T]\to \mathbb{R}$ denote a positive function that is equal to $p:{\mathbb{R}}^{d}\to \mathbb{R}$ on ∂Ω . Note that we can assume without loss of generality that p is positive on ∂Ω for t>0 . This yields the forward Fokker–Planck equation (2.14) on the domain Ω with initial data $p_{0}$ restricted to Ω and Dirichlet boundary conditions $p_{B}$ on ∂Ω×[0,T] . In addition, we set $u_{B}=\log\;p_{B}$ on ∂Ω×[0,T] .

The density $p_{\theta }^{\text{SDE}}$ of the approximate SDE equation (2.7) also satisfies a forward Fokker–Planck equation, which can be derived by writing the Fokker–Planck equation for the forward dynamics equation (2.8) of $p_{\theta }^{\text{SDE}}$ with appropriate terminal distribution. This gives the *approximate Fokker–Planck equation* (in forward time):


(2.15) $$\begin{array}{r} \frac{\partial p_{\theta }^{\text{SDE}}}{\partial t}(x,t)+\nabla \cdot (f_{\theta }^{\text{SDE}}(x,t)p_{\theta }^{\text{SDE}}(x,t))+\frac{1}{2}g^{2}(t)\nabla^{2}(p_{\theta }^{\text{SDE}}(x,t))=0 \end{array}$$

on ${\mathbb{R}}^{d}\times \left[0,T\right]$ , equipped with the terminal condition π from assumptions 2.1 on the full space ${\mathbb{R}}^{d}$ , i.e. $p_{\theta }^{\text{SDE}}(x,T)=\pi (x)$ for all $x\in {\mathbb{R}}^{d}$ , where π is typically specified as a Gaussian approximation of p(⋅,T) . For considering equation (2.15) on a bounded domain Ω , we introduce positive Dirichlet boundary conditions. Let $p_{B}^{\text{SDE}}:\partial \Omega \times [0,T]\to R$ denote a positive function that is equal to $p_{\theta }^{\text{SDE}}:R^{d}\to R$ on ∂Ω . We obtain the approximate Fokker–Planck equation equation (2.15) on the domain Ω with initial data $p_{0}$ restricted to Ω and Dirichlet boundary conditions $p_{B}^{\text{SDE}}$ on ∂Ω×[0,T] . We set $u_{B}^{\text{SDE}}=\log p_{B}^{\text{SDE}}$ on ∂Ω×[0,T] .

Note that for $p_{\theta }^{\text{SDE}}$ , we always assume a fixed terminal condition at time T when considering equation (2.15) in t (or an initial condition when considering the evolution in reverse time τ ) as $p_{\theta }^{\text{SDE}}$ describes the flow of probability backwards from a Gaussian approximation π of p(x,T) to some approximation of $p_{0}$ . Notice that equations (2.14) and (2.15) are of a similar form, apart from the different signs of the diffusion terms.

In addition to considering Fokker–Planck equations for the densities, one can also introduce log-Fokker–Planck equations for the potential. For the density p satisfying the forward Fokker–Planck equation (2.14) for the forward SDE equation (2.1) and the associated potential u=log⁡p we introduce the *forward log-Fokker–Planck equation* (in forward time) as


(2.16) $$\begin{array}{rl} \frac{\partial u}{\partial t}(x,t)+\nabla \cdot f(x,t) & +\nabla u(x,t)\cdot f(x,t)-\frac{1}{2}g^{2}(t)\|\nabla u(x,t){\|}_{2}^{2}-\frac{1}{2}g^{2}(t)\nabla^{2}u(x,t)=0 \end{array}$$

on ${\mathbb{R}}^{d}\times \left[0,T\right]$ . On the domain Ω , we equip equation (2.14) with initial data $u_{0}$ restricted to Ω and boundary conditions $u_{B}$ on ∂Ω×[0,T] .

For $p_{\theta }^{\text{SDE}}$ solving equation (2.15) in forward time, the log-density $u_{\theta }^{\text{SDE}}=\log p_{\theta }^{\text{SDE}}$ satisfies the *approximate log-Fokker–Planck equation* (in forward time) given by


(2.17) $$\begin{array}{r} \begin{array}{rl} \frac{\partial u_{\theta }^{\text{SDE}}}{\partial t}(x,t)+\nabla \cdot f_{\theta }^{\text{SDE}}(x,t) & +\nabla u_{\theta }^{\text{SDE}}(x,t)\cdot f_{\theta }^{\text{SDE}}(x,t)\\ & \;+\frac{1}{2}g^{2}(t)\|\nabla u_{\theta }^{\text{SDE}}(x,t){\|}_{2}^{2}+\frac{1}{2}g^{2}(t)\nabla^{2}u_{\theta }^{\text{SDE}}(x,t)=0 \end{array} \end{array}$$

on ${\mathbb{R}}^{d}\times \left[0,T\right]$ . On the domain Ω , we again equip equation (2.17) with terminal data $u_{T}$ restricted to Ω and boundary conditions $u_{B}^{\text{SDE}}$ on ∂Ω×[0,T] .

### (f) Fokker–Planck residuals

Our analysis in §3 is concerned with quantifying how the consistency of the neural approximation with the underlying Fokker–Planck equations is related to the ODE–SDE gap observed in practice. To measure this consistency, we derive a residual for the log-Fokker–Planck equation governing the evolution of the potential. In our theory, we use the residual as an error measure, and in our numerics, we add it as a regularization term to couple with the denoising score-matching objective $L_{DSM}$ in equation (2.5). Details on the implementation are given in §4.

Restricting ourselves to a bounded domain $\Omega \subset {\mathbb{R}}^{d}$ with $\partial \Omega \in C^{\infty }$ , we consider a neural approximation $u_{\theta }$ with parameters θ , which learns the solution $u_{\theta }^{\text{SDE}}$ of the approximate log-Fokker–Planck equation (2.17) such that $u_{\theta }$ satisfies appropriate Dirichlet boundary conditions $p_{B}^{\text{SDE}}$ and terminal condition $p_{\theta }(\cdot ,T)=\pi$ . This boundary condition ensures that the solution to equation (2.17) on Ω is everywhere equal to its corresponding solution on an unbounded domain.

Note that setting $u_{\theta }=u_{\theta }^{\text{SDE}}$ in equation (2.6) results in the probability flow ODE of the approximate SDE equation (2.9) (with density $p_{\theta }^{\text{SDE}}$ ) and approximate probability flow ODE equation (2.10) (with density $p_{\theta }^{ODE}$ ) coinciding. Thus, the gap between these two generative processes relates to the consistency of our neural approximation with the log-Fokker–Plank equation (2.17) associated with the approximate SDE. To measure the Fokker–Planck consistency, we substitute our model $u_{\theta }$ into equation (2.17) and measure the differential operator residual in the $L^{2}$ -norm. Manipulating this residual, we obtain


(2.18) $$\begin{array}{r} \begin{array}{rl} & \frac{\partial u_{\theta }}{\partial t}(x,t)+\nabla \cdot f_{\theta }^{\text{SDE}}(x,t)+\nabla u_{\theta }(x,t)\cdot f_{\theta }^{\text{SDE}}(x,t)+\frac{1}{2}g^{2}(t)\|\nabla u_{\theta }(x,t){\|}_{2}^{2}+\frac{1}{2}g^{2}(t)\nabla^{2}u_{\theta }(x,t)\\ & \equiv \frac{\partial u_{\theta }}{\partial t}(x,t)+\nabla \cdot (f(x,t)-g^{2}(t)\nabla u_{\theta }(x,t))+\nabla u_{\theta }(x,t)\cdot (f(x,t)-g^{2}(t)\nabla u_{\theta }(x,t))\\ & \;+\frac{1}{2}g^{2}(t)\|\nabla u_{\theta }(x,t){\|}_{2}^{2}+\frac{1}{2}g^{2}(t)\nabla^{2}u_{\theta }(x,t)\\ & \equiv \frac{\partial u_{\theta }}{\partial t}(x,t)+\nabla \cdot f(x,t)+\nabla u_{\theta }(x,t)\cdot f(x,t)-\frac{1}{2}g^{2}(t)\|\nabla u_{\theta }(x,t){\|}_{2}^{2}-\frac{1}{2}g^{2}(t)\nabla^{2}u_{\theta }(x,t) \end{array} \end{array}$$

on Ω×[0,T] . This demonstrates that the residual for the forward log-Fokker–Planck equation (2.16) is equivalent to the residual for the approximate log-Fokker–Planck equation (2.17) , thus it is sufficient to only consider the residual of the forward equation. Hence, we define the residual of the log-Fokker–Planck equation for the approximate reverse SDE equation (2.7) for any t∈[0,T) as


(2.19) $$\begin{array}{r} \begin{array}{rl} \tilde{R}(\theta ,u_{\theta },t) & =V(T-t{)}^{-1}\int_{t}^{T}\|\frac{\partial u_{\theta }}{\partial s}(\cdot ,s)+\nabla \cdot f(\cdot ,s)+\nabla u_{\theta }(\cdot ,s)\cdot f(\cdot ,s)\\ & \;\;\;{-\frac{1}{2}g^{2}(s)\|\nabla u_{\theta }(\cdot ,s){\|}_{2}^{2}-\frac{1}{2}g^{2}(s)\nabla^{2}u_{\theta }(x,s)\|}_{L^{2}(\Omega )}^{2}ds, \end{array} \end{array}$$

where V(r):=rVol⁡(Ω) is the volume of [T−r,T]×Ω . We refer to $\tilde{R}$ as the *log-Fokker–Planck residual*. This residual quantifies how well our approximation $u_{\theta }$ agrees with the true solution $u_{\theta }^{\text{SDE}}$ to the approximate log-Fokker–Planck equation. In §3, we show how the values attained by this residual define an upper bound on the ODE–SDE discrepancy.

Note that analogous calculations hold for the residual in the standard Fokker–Planck equation (2.15) , and the score-Fokker–Planck equation discussed in §3c. In all cases, the residual corresponding to their forward Fokker–Planck equations is equal to the residual of the Fokker–Planck equation of the generative process, and these residuals relate to the ODE–SDE gap.

We remark that the bounded domain assumption made at the beginning of this subsection arises from the PDE analysis we apply to relate the neural approximation to the Fokker–Planck equation. This contrasts with other works deriving bounds for diffusion models since they do not investigate this relation, typically only considering generation under an $L^{2}$ -score approximation. In practice, this assumption is not restrictive, since for any data distribution $p_{0}$ and ϵ>0 it is possible to choose a bounded domain Ω such $\int_{\Omega }p_{\theta }(x,t)dx>1-\epsilon$ for all times t∈[0,T] . Therefore, the chance that a trajectory escapes this domain in practice is diminished.

## 3. Theoretical results on the ODE–SDE gap

In this section, we investigate the gap between the ODE- and SDE-induced distributions in terms of Fokker–Planck equations. More precisely, we derive bounds related to the approximate log-Fokker–Planck equation (2.17) in §3a, and in §3b we show that this theory applies to the associated potential model. In §3c, we provide a sketch of how to derive analogous bounds in terms of the approximate score-Fokker–Planck equation, thus addressing the common score parameterization. All these results are based on the following:

**Assumptions 3.1.** *Let* t∈[0,T) *and* δ>0 *be given, let* $\Omega \subset {\mathbb{R}}^{d}$ *be a bounded domain with* $\partial \Omega \in C^{\infty }$ . *Assume that* $p_{\theta }^{\text{SDE}}$ *satisfies* *equation (2.15)* *on* Ω *with terminal condition* π *restricted to* Ω *and Dirichlet boundary conditions* $p_{B}^{\text{SDE}}$ *on* ∂Ω×[0,T] *, with* $u_{\theta }^{\text{SDE}}=\log p_{\theta }^{\text{SDE}}$ . *Further, let* $p_{\theta }^{ODE}$ *be the probability density associated with* *equation (2.10)* *with terminal condition* π *restricted to* Ω .

### (a) The ODE–SDE gap for the approximate Fokker–Planck equation

We show that, at a fixed time t∈[0,T] *,* $p_{\theta }^{\text{SDE}}(\cdot ,t)$ satisfying the approximate Fokker–Planck equation equation (2.15) converges to the density $p_{\theta }^{ODE}(\cdot ,t)$ of the approximate probability flow ODE equation (2.10) with respect to the Wasserstein 2-distance $W_{2}$ as the log-Fokker–Planck residual $\tilde{R}(\theta ,u_{\theta },t)$ in equation (2.19) goes to zero

**Theorem 3.1.** *Assume that assumptions* 2.1 *and* 3.1 *hold. Further, assume that the neural network* $u_{\theta }\in C^{\infty }(\Omega \times [0,T])$ *is determined such that* $u_{\theta }$ *obeys the terminal condition* $u_{T}=\log\;\pi$ *restricted to* Ω *and Dirichlet boundary conditions* $u_{B}^{\text{SDE}}$ *on* ∂Ω×[0,T] *, and* $\tilde{R}(\theta ,u_{\theta },t)$ *in* (2.19) *satisfies* $\tilde{R}(\theta ,u_{\theta },t)<\delta$ . *Then,* $W_{2}(p_{\theta }^{ODE}(\cdot ,t),p_{\theta }^{\text{SDE}}(\cdot ,t))<C\delta$ *for some constant* C>0 *independent of* δ .

We provide the proof of theorem 3.1 in appendix A. The constant C in theorem 3.1 depends on the time horizon T as well as the Lipschitz constants of $\nabla u_{\theta }$ and $\nabla u_{\theta }^{\text{SDE}}$ . Details of these dependencies are specified in the proof.

### (b) The ODE–SDE gap for the potential model associated with the approximate Fokker–Planck equation

A key benefit of score-based models is that scores are agnostic to multiplicative scaling of the underlying density, implying known normalizing constants are not required for their implementation. So far, we have implicitly assumed that $u_{\theta }$ is an approximation of $\log(p_{\theta })$ for a density $p_{\theta }$ , and hence that the integral of $\exp(u_{\theta })$ is normalized which is non-trivial in practice. To overcome this issue, we introduce an unnormalized network $\phi_{\theta }$ as a potential model and relate it to $u_{\theta }$ by introducing a (potentially time-varying) normalizing constant $Z_{t}$ for $\exp(\phi_{\theta }(\cdot ,t))$ . This gives the relation


(3.1) $$\begin{array}{r} \phi_{\theta }(x,t)=u_{\theta }(x,t)+\log Z_{t}, \end{array}$$

which we also use to obtain the terminal data $\phi_{T}$ and boundary conditions $\phi_{B}^{\text{SDE}}$ on ∂Ω×[0,T] . The bounds on the ODE–SDE gap in theorem 3.1 also hold when considering $\phi_{\theta }$ instead of $u_{\theta }$ in the Fokker–Planck residual equation (2.19), as shown in appendix B.

### (c) The ODE–SDE gap for the approximate score-Fokker–Planck equation

In this work, we primarily focus on the underlying connection between the ODE, the SDE and Fokker–Planck equations observed in score-based diffusion models, and thus our focus has been on the potential parameterization discussed so far. However, in most practical implementations, a score parameterization is adopted due to computational efficiency, given by $s_{\theta }(x,t)\approx \nabla u(x,t)=\nabla \log\;p(x,t)$ for the density p and the log-density u of the forward SDE equation (2.1). The score parameterization is linked to the score-Fokker–Planck equation, see e.g.[12]. To ensure applicability of our results to this case, we argue in this section that the bounds on the ODE–SDE gap in theorem 3.1 also hold for the score parameterization.

A score-Fokker–Planck equation can be derived by taking the gradient of the associated log-Fokker–Planck equation. To derive an analogous result to theorem 3.1, we are interested in the score-Fokker–Planck equation of the approximate SDE with log-Fokker–Planck equation (2.17) . Taking the gradient of equation (2.17) and setting $s_{\theta }^{\text{SDE}}(x,t)=\nabla u_{\theta }^{\text{SDE}}(x,t)\in R^{d}$ yields the *approximate score-Fokker–Planck equation*:


(3.2) $$\begin{array}{r} \begin{array}{rl} \frac{\partial s_{\theta }^{\text{SDE}}}{\partial t}(x,t) & +\nabla (\nabla \cdot f_{\theta }^{\text{SDE}}(x,t))+(\nabla s_{\theta }^{\text{SDE}}(x,t))f_{\theta }^{\text{SDE}}(x,t)+(\nabla f_{\theta }^{\text{SDE}}(x,t))s_{\theta }^{\text{SDE}}(x,t)\\ & +g^{2}(t)(\nabla s_{\theta }^{\text{SDE}}(x,t))s_{\theta }^{\text{SDE}}(x,t)+\frac{1}{2}g^{2}(t)\nabla (\nabla \cdot s_{\theta }^{\text{SDE}}(x,t))=0. \end{array} \end{array}$$

Here, we use analogous notation to the potential case so that $s_{\theta }^{\text{SDE}}(x,t)$ is the true score associated with the approximate reverse SDE equation (2.7) and is linked with the density $p_{\theta }^{\text{SDE}}$ via $s_{\theta }^{\text{SDE}}=\nabla \log p_{\theta }^{\text{SDE}}$ . Similarly, we also introduce the score $s_{\theta }^{ODE}(x,t)=\nabla u_{\theta }^{ODE}(x,t)=\nabla \log\;p_{\theta }^{ODE}(x,t)\in {\mathbb{R}}^{d}$ associated with the approximate probability flow ODE equation (2.10). As before, we consider appropriate terminal data and Dirichlet boundary conditions.

Let $s_{\theta }(x,t)$ denote a score model approximating ∇log⁡p(x,t) . Following an analogous calculation to equation (2.18), the residual corresponding to the approximate score-Fokker–Planck equation (3.2) can be written as the residual of a score-Fokker–Planck equation, and we define the *score-Fokker–Planck residual* by


(3.3) $$\begin{array}{r} \begin{array}{rl} R^{s}(\theta ,s_{\theta },t) & =V(T-t{)}^{-1}\int_{t}^{T}\|\frac{\partial s_{\theta }}{\partial r}(\cdot ,r)+\nabla (\nabla \cdot f(\cdot ,r))+(\nabla s_{\theta }(\cdot ,r))f(\cdot ,r)\\ & \;\;{+(\nabla f(\cdot ,r))s_{\theta }(\cdot ,r)-g^{2}(r)(\nabla s_{\theta }(\cdot ,r))s_{\theta }(\cdot ,r)-\frac{1}{2}g^{2}(r)\nabla (\nabla \cdot s_{\theta }(\cdot ,r))\|}_{L^{2}(\Omega )}^{2}dr. \end{array} \end{array}$$

We can now state analogous result to theorem 3.1 for the score-Fokker–Planck equation:

**Theorem 3.2.** *Assume that assumptions* 2.1 *and* 3.1 *hold. Further, assume that* $s_{\theta }\in C^{\infty }(\Omega \times [0,T])$ *is determined such that* $R^{s}(\theta ,s_{\theta },t)<\delta$ *and equipped with appropriate terminal and Dirichlet boundary conditions. Then,* $W_{2}(p_{\theta }^{ODE}(\cdot ,t),p_{\theta }^{\text{SDE}}(\cdot ,t))<C\delta$ *for some* C>0 *independent of* δ .

Theorem 3.2 can be derived by applying analogous Steps I and II in the proof of theorem 3.1 and generalizing them to vector-valued functions as appropriate. More precisely, Step I of the proof has to be generalized to vector-valued solutions $s_{\theta }^{\text{SDE}}$ of equation (3.2) as opposed to the scalar solution $u_{\theta }^{\text{SDE}}$ of equation (2.17) but due to the similarity of the equations, this step can be done analogously. Step II follows as in the proof of theorem 3.1. Due to the similarity of the proofs, the detailed proof is omitted here.

## 4. Numerical experiments

To demonstrate our analytical results numerically and ensure visual interpretability, we implement several diffusion models in ${\mathbb{R}}^{2}$ that attain a range of log-Fokker–Planck residual values (equation (2.19)) using various toy datasets. For the forward SDE, we choose f(x,t)=−x and g(t)=1 , resulting in the simple Ornstein–Uhlenbeck process $dx_{t}=-x_{t}dt+dW_{t}.$ Following equation (2.16), the associated log-Fokker–Planck equation is given by $\partial u(x,t)/\partial t=2+x\cdot \nabla u(x,t)+\frac{1}{2}\|\nabla u(x,t){\|}_{2}^{2}+\frac{1}{2}\nabla^{2}u(x,t).$ In our experiments, we take three different data distributions and train a neural network to minimize the loss function $L(\theta ,w_{R}):=L_{DSM}(\theta ,\nabla \phi_{\theta },\lambda )+w_{R}\tilde{R}(\theta ,\phi_{\theta },0)$ for differing values of $w_{R}$ , where $L_{DSM}$ and $\tilde{R}$ are defined in equations (2.5) and equation (2.19), respectively, and λ is set according to the likelihood weighting. Note that for our specific setting, we have


$$\begin{array}{r} \tilde{R}\left(\theta ,\phi_{\theta },t\right)=E_{\begin{array}{c} s∼U(t,T)\\ x∼U(\Omega ) \end{array}}\left[{\left(\frac{\partial \phi_{\theta }(x,s)}{\partial t}-2-x\cdot \nabla \phi_{\theta }(x,s)-\frac{1}{2}{\left\|\nabla \phi_{\theta }(x,s)\right\|}_{2}^{2}-\frac{1}{2}\nabla^{2}\phi_{\theta }(x,s)\right)}^{2}\right], \end{array}$$

**Figure 1.**
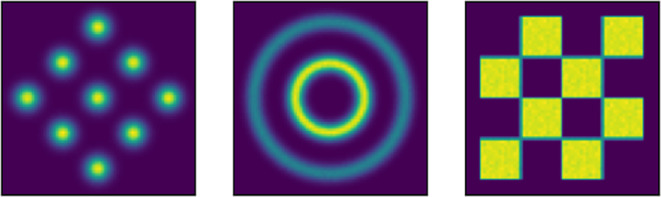
Left to right our three examples are a Gaussian mixture, a concentric circles distribution and a checkerboard distribution.

Therefore, both the denoising score matching objective $L_{DSM}(\theta ,\nabla \phi_{\theta },\lambda )$ and the log-Fokker–Planck residual $\tilde{R}(\theta ,\phi_{\theta },0)$ are approximated using Monte Carlo estimation. We set T=10 and the likelihood weighting implies λ(t)=1 for t∈[0,T] . Note that we do not add terms that enforce boundary conditions in space or time, since the denoising score matching objective (2.5) already encourages consistency with these conditions (up to a multiplicative constant proportional to the underlying density). We generate samples from $p_{\theta }^{\text{SDE}}(\cdot ,0)$ and $p_{\theta }^{ODE}(\cdot ,0)$ using Euler–Maruyama and Euler discretizations of the reverse approximate SDE equation (2.7) and the reverse approximate probability flow ODE equation (2.10), respectively. To validate our results, we generate three million samples from each distribution. Due to computational constraints, these samples are then discretized on to a 64×64 grid. When computing the Wasserstein distances to the target distribution and producing visualizations, we will consider the discretized distributions. Figure 1 shows the target distributions for our experiments.

We choose two-dimensional examples to ensure we can explicitly see the behaviour of the distributions and measure $W_{2}$ distances. Our examples cover the analytically solvable Gaussian mixture case, a smooth concentric circles distribution and a discontinuous checkerboard distribution to cover a range of scenarios of potential interest.

**Figure 2.**
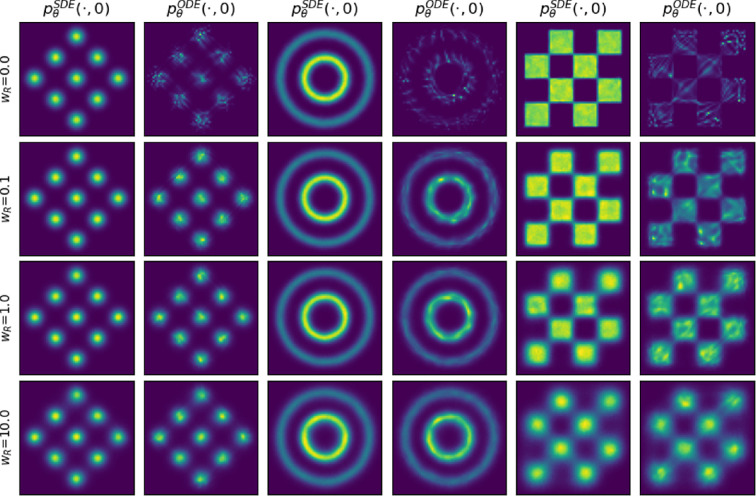
Distributions of $p_{\theta }^{\text{SDE}}(\cdot ,0)$ and $p_{\theta }^{ODE}(\cdot ,0)$ for weighting parameters $w_{R}$ taking values in (0,0.1,1,10) . The rows indicate which weighting parameter was used, while the columns indicate whether the displayed distribution is of $p_{\theta }^{\text{SDE}}(\cdot ,0)$ or of $p_{\theta }^{ODE}(\cdot ,0)$ in the corresponding experiment. Samples displayed from the ODE and SDE samplers were attained using the same score model.

We parameterize our potential model $\phi_{\theta }(x,t)$ by a fully connected neural network with two hidden layers of 80 nodes. We apply softplus activation functions, which have well defined first and second derivatives as required to evaluate $\tilde{R}(\theta ,\phi_{\theta },t)$ . Each model is trained for 100 000 iterations using Adam with learning rate decaying from $10^{-3}$ down to $10^{-5}$ . Figure 2 shows the samples obtained from $p_{\theta }^{\text{SDE}}(\cdot ,0)$ and $p_{\theta }^{ODE}(\cdot ,0)$ for different weighting parameters $w_{R}$ .

**Figure 3.**
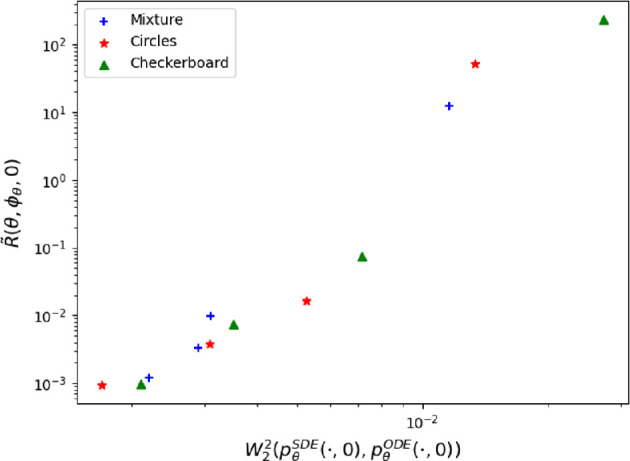
Comparison of the log-Fokker–Planck residual values against the Wasserstein distance between the ODE and SDE samples.

**Figure 4.**
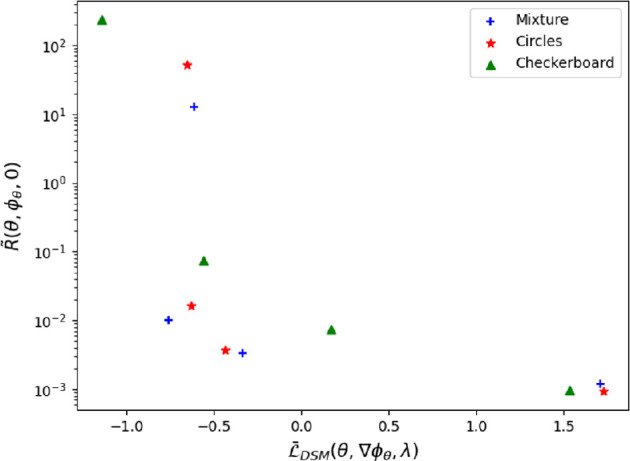
Comparison of the log-Fokker–Planck residual values against the denoising score matching loss. We see that the attainment of a low $\tilde{R}$ is correlated with a higher $L_{DSM}$ , thus explaining the degradation in sample quality for high $w_{R}$ . Here, the $L_{DSM}$ have been normalized by data distribution to eliminate bias specific to each dataset.

**Figure 5.**
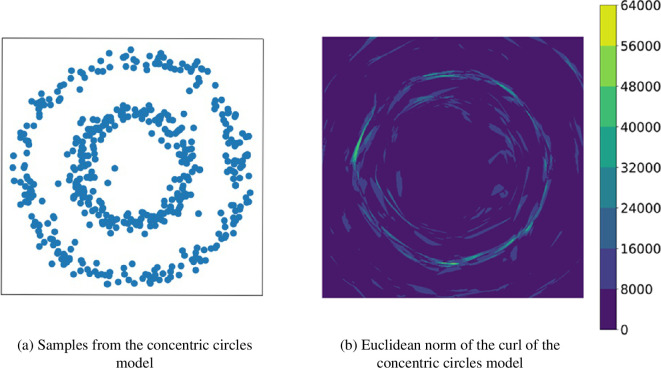
Samples and Euclidean norm of the curl of a trained (non-potential) score model (i.e. $\|\nabla \times s_{\theta }(0,x){\|}_{2}$ ). Clearly, the score model $s_{\theta }(0,x)$ is not a conservative vector field.

We see from figure 2 that if we only optimize $L_{DSM}$ (i.e. for $w_{R}=0$ ) the resulting $p_{\theta }^{ODE}(\cdot ,0)$ is quite different from the true distribution. Notably, areas of high probability in $p_{\theta }^{ODE}(\cdot ,0)$ do coincide with high probability regions of $p_{\theta }^{\text{SDE}}(\cdot ,0)$ . Therefore in typical generative modelling scenarios, it may be difficult to identify this mischaracterization of the data distribution, given that individual samples generated from $p_{\theta }^{ODE}(\cdot ,0)$ are generally plausible. Visually, we see that adding a factor of $\tilde{R}(\theta ,\phi_{\theta },0)$ to the loss function initially results in an improvement in $p_{\theta }^{ODE}$ . The tables and figures inappendix C further detail these results. In table 2, we see that the distance between $p_{\theta }^{ODE}(\cdot ,0)$ and $p_{0}$ consistently reduces for $w_{R}=0.1$ and $w_{R}=1$ when compared with $w_{R}=0$ , indicating an improvement in ODE samples. Increasing $w_{R}$ beyond this further reduces the gap between $p_{\theta }^{ODE}(\cdot ,0)$ and $p_{\theta }^{\text{SDE}}(\cdot ,0)$ as listed in table 1; however, this comes at the cost of increasing the distance from both $p_{\theta }^{ODE}(\cdot ,0)$ and $p_{\theta }^{\text{SDE}}(\cdot ,0)$ to $p_{0}$ which can be observed in tables 2 and 3 for $w_{R}=10$ . This can clearly be seen in figure 2 by the overly smoothed distributions that are attained with higher $w_{R}$ . Table 3 lists that the quality of $p_{\theta }^{\text{SDE}}$ degrades monotonically with increasing $w_{R}$ , which results in the negative correlation between $\tilde{R}(\theta ,\phi_{\theta },0)$ and $L_{DSM}(\theta ,\nabla \phi_{\theta },\lambda )$ observed in figure 4. From this, we conclude that the cost of improving $p_{\theta }^{ODE}$ is a reduction in the quality of $p_{\theta }^{\text{SDE}}$ . Finally, in figure 3, we evaluate $\tilde{R}(\theta ,\phi_{\theta },0)$ for each of our trained models and visualize the relation between $\tilde{R}(\theta ,\phi_{\theta },0)$ and the associated $W_{2}(p_{\theta }^{ODE}(\cdot ,0),p_{\theta }^{\text{SDE}}(\cdot ,0))$ values. This demonstrates a clear positive correlation supporting our theoretical analysis, where we proved an upper bound on the Wasserstein 2-distance between the ODE- and SDE-induced distributions in terms of a log-Fokker–Planck residual $\tilde{R}$ .

## 5. Conclusions

In this work, we conducted a systematic investigation into the dynamics that arise in score-based diffusion models. We mainly focused on the differences between the generative densities $p_{\theta }^{\text{SDE}}$ and $p_{\theta }^{ODE}$ defined by the reverse approximate SDE and the approximate probability flow ODE, respectively. Analytically, we proved that the discrepancy between $p_{\theta }^{\text{SDE}}$ and $p_{\theta }^{ODE}$ can be bounded by a log-Fokker–Planck residual in the Wasserstein 2-distance, thus giving a deeper insight into the connection between the two generative distributions in terms of the Fokker–Planck dynamics underlying the diffusion process. Numerically, we showed that $p_{\theta }^{\text{SDE}}$ and $p_{\theta }^{ODE}$ can differ substantially when the neural network is trained using the standard score-matching objective. Our numerical experiments also demonstrate that penalizing the loss function by the log-Fokker–Planck residual indeed leads to closing the gap between the ODE and the SDE distributions in the Wasserstein 2-distance. Our findings revealed that imposing this additional constraint within our loss function could improve the quality of $p_{\theta }^{ODE}$ when compared with the ground truth, though in exchange for this we observed concurrent degradation in the quality of $p_{\theta }^{\text{SDE}}$ . The practical implication of these findings is that enforcing self-consistency through penalization by the log-Fokker–Planck residual is unlikely to improve state-of-the-art generation using stochastic samplers. However, for downstream tasks where deterministic generation is required, such penalization could provide a potential avenue to improve sample quality and likelihood accuracy.

## Data Availability

The code and data to reproduce the results is available at https://github.com/teojd/ODESDEGap/tree/main. They are also available on Zenodo: https://zenodo.org/records/15303316.

## Appendix A. Proof of the ODE-SDE gap for the approximate Fokker–Planck equation

*Proof of theorem* 3.1. We proceed in two steps. First, we show that there exists a constant $\tilde{C}>0$ independent of δ such that $\|u_{\theta }(\cdot ,\tau )-u_{\theta }^{\text{SDE}}(\cdot ,\tau ){\|}_{L^{2}(\Omega )}<\tilde{C}\delta$ which allows us to show that $\int_{0}^{\tau }\|\nabla u_{\theta }(\cdot ,s)-\nabla u_{\theta }^{\text{SDE}}(\cdot ,s){\|}_{L^{2}(\Omega )}^{2}ds<C\delta$ for the reverse time variable τ=T−t∈(0,T] where the constant C>0 is independent of δ . Finally, we prove that

$$W_{2}(p_{\theta }^{ODE}(\cdot ,t),p_{\theta }^{\text{SDE}}(\cdot ,t))<C\delta$$

for some constant C>0 independent of δ .

**Step I:** To prove the first part, assume that $u_{\theta }^{\text{SDE}}$ solves equation (2.17) on Ω×[0,T] *,* that is,

(A 1)
$$\begin{array}{r} \begin{array}{rl} \frac{\partial u_{\theta }^{\text{SDE}}}{\partial t}(x,t) & +\nabla \cdot f_{\theta }^{\text{SDE}}(x,t)+\nabla u_{\theta }^{\text{SDE}}(x,t)\cdot f_{\theta }^{\text{SDE}}(x,t)\\ & +\frac{1}{2}g^{2}(t)\|\nabla u_{\theta }^{\text{SDE}}(x,t){\|}_{2}^{2}+\frac{1}{2}g^{2}(t)\nabla^{2}u_{\theta }^{\text{SDE}}(x,t)=0, \end{array} \end{array}$$

for (x,t)∈Ω×[0,T] *,* equipped with terminal data $u_{T}$ restricted to Ω and boundary conditions $u_{B}^{\text{SDE}}$ on ∂Ω×[0,T] . Further, assume that $u_{\theta }$ satisfies equation (2.17) on Ω×[0,T] with some residual q , i.e.

(A 2)
$$\begin{array}{r} \begin{array}{rl} \frac{\partial u_{\theta }}{\partial t}(x,t) & +\nabla \cdot f_{\theta }^{\text{SDE}}(x,t)+\nabla u_{\theta }(x,t)\cdot f_{\theta }^{\text{SDE}}(x,t)\\ + & \frac{1}{2}g^{2}(t)\|\nabla u_{\theta }(x,t){\|}_{2}^{2}+\frac{1}{2}g^{2}(t)\nabla^{2}u_{\theta }(x,t)=q(x,t), \end{array} \end{array}$$

for (x,t)∈Ω×[0,T] *,* equipped with terminal data $u_{T}$ restricted to Ω and boundary conditions $u_{B}^{\text{SDE}}$ on ∂Ω×[0,T] .

As equations (A 1) and (A 2) are equipped with terminal conditions, it is more natural to work with the corresponding reverse time equations. Writing the reverse time variable as τ=T−t∈[0,T] we have

(A 3)
$$\begin{array}{r} \begin{array}{rl} \frac{\partial {\bar{u}}_{\theta }^{\text{SDE}}}{\partial \tau }(x,\tau ) & -\nabla \cdot {\bar{f}}_{\theta }^{\text{SDE}}(x,\tau )-\nabla {\bar{u}}_{\theta }^{\text{SDE}}(x,\tau )\cdot {\bar{f}}_{\theta }^{\text{SDE}}(x,\tau )\\ & -\frac{1}{2}{\bar{g}}^{2}(\tau )\|\nabla {\bar{u}}_{\theta }^{\text{SDE}}(x,\tau ){\|}_{2}^{2}-\frac{1}{2}{\bar{g}}^{2}(\tau )\nabla^{2}{\bar{u}}_{\theta }^{\text{SDE}}(x,\tau )=0 \end{array} \end{array}$$

and

(A 4)
$$\begin{array}{r} \begin{array}{rl} \frac{\partial {\bar{u}}_{\theta }}{\partial \tau }(x,\tau ) & -\nabla \cdot {\bar{f}}_{\theta }^{\text{SDE}}(x,\tau )-\nabla {\bar{u}}_{\theta }(x,\tau )\cdot {\bar{f}}_{\theta }^{\text{SDE}}(x,\tau )\\ & -\frac{1}{2}{\bar{g}}^{2}(\tau )\|\nabla {\bar{u}}_{\theta }(x,\tau ){\|}_{2}^{2}-\frac{1}{2}{\bar{g}}^{2}(\tau )\nabla^{2}{\bar{u}}_{\theta }(x,\tau )=\bar{q}(x,\tau ). \end{array} \end{array}$$

Note that the log-Fokker–Planck residual equation (2.19) is related to $\bar{q}(\cdot ,\tau )$ for τ=T−t by

(A 5)
$$\begin{array}{r} \begin{array}{r} \tilde{R}(\theta ,u_{\theta },t)=\tilde{R}(\theta ,u_{\theta },T-\tau )=V(\tau {)}^{-1}\int_{T-\tau }^{T}\|q(\cdot ,s){\|}_{L^{2}(\Omega )}^{2}ds=V(\tau {)}^{-1}\int_{0}^{\tau }\|\bar{q}(\cdot ,s){\|}_{L^{2}(\Omega )}^{2}ds, \end{array} \end{array}$$

which follows from equation (2.18) and (A 2). Subtracting equation (A 3) for ${\bar{u}}_{\theta }^{\text{SDE}}$ from equation (A 4) for ${\bar{u}}_{\theta }$ yields

$$\begin{array}{r} \begin{array}{rl} & \frac{\partial ({\bar{u}}_{\theta }(x,\tau )-{\bar{u}}_{\theta }^{\text{SDE}}(x,\tau ))}{\partial \tau }(x,\tau )-\nabla ({\bar{u}}_{\theta }(x,\tau )-{\bar{u}}_{\theta }^{\text{SDE}}(x,\tau ))\cdot {\bar{f}}_{\theta }^{\text{SDE}}(x,\tau )\\ & \;-\frac{1}{2}{\bar{g}}^{2}(\tau )(\|\nabla {\bar{u}}_{\theta }(x,\tau ){\|}_{2}^{2}-\|\nabla {\bar{u}}_{\theta }^{\text{SDE}}(x,\tau ){\|}_{2}^{2})-\frac{1}{2}{\bar{g}}^{2}(\tau )\nabla^{2}({\bar{u}}_{\theta }-{\bar{u}}_{\theta }^{\text{SDE}})(x,\tau )=\bar{q}(x,\tau ). \end{array} \end{array}$$

We define the error $e_{\bar{u}}={\bar{u}}_{\theta }-{\bar{u}}_{\theta }^{\text{SDE}}$ on Ω×[0,T] . Note that the boundary and terminal conditions of ${\bar{u}}_{\theta }^{\text{SDE}}$ and ${\bar{u}}_{\theta }$ imply that $e_{\bar{u}}(x,0)=0$ for all x∈Ω and that $e_{\bar{u}}$ has homogeneous Dirichlet boundary conditions. This allows us to write a PDE for the error $e_{\bar{u}}$ given by

$$\begin{array}{rl} \frac{\partial e_{\bar{u}}}{\partial \tau }(x,\tau )-\nabla e_{\bar{u}}(x,\tau )\cdot {\bar{f}}_{\theta }^{\text{SDE}}(x,\tau )-\frac{1}{2}{\bar{g}}^{2}(\tau )(\|\nabla {\bar{u}}_{\theta }(x,\tau ){\|}_{2}^{2} & -\|\nabla {\bar{u}}_{\theta }^{\text{SDE}}(x,\tau ){\|}_{2}^{2})\\ & -\frac{1}{2}{\bar{g}}^{2}(\tau )\nabla^{2}e_{\bar{u}}(x,\tau )=\bar{q}(x,\tau ). \end{array}$$

Using $\frac{1}{2}\frac{\partial e_{\bar{u}}^{2}}{\partial \tau }=e_{\bar{u}}\frac{\partial e_{\bar{u}}}{\partial \tau }$ , we obtain an equation for the squared error $e_{\bar{u}}^{2}$ that reads

$$\begin{array}{r} \begin{array}{rl} \frac{1}{2}\frac{\partial e_{\bar{u}}^{2}}{\partial \tau }(x,\tau )=e_{\bar{u}}(x,\tau )\nabla e_{\bar{u}}(x,\tau ) & \cdot {\bar{f}}_{\theta }^{\text{SDE}}(x,\tau )+\frac{1}{2}{\bar{g}}^{2}(\tau )e_{\bar{u}}(x,\tau )(\|\nabla {\bar{u}}_{\theta }(x,\tau ){\|}_{2}^{2}-\|\nabla {\bar{u}}_{\theta }^{\text{SDE}}(x,\tau ){\|}_{2}^{2})\\ & +\frac{1}{2}{\bar{g}}^{2}(\tau )e_{\bar{u}}(x,\tau )\nabla^{2}(e_{\bar{u}}(x,\tau ))+e_{\bar{u}}(x,\tau )\bar{q}(x,\tau ). \end{array} \end{array}$$

We integrate to get an equation for the $L^{2}$ -error given by

$$\begin{array}{r} \begin{array}{rl} \frac{1}{2}\frac{\partial }{\partial \tau }\|e_{\bar{u}}(\cdot ,\tau ){\|}_{L^{2}(\Omega )}^{2} & =\int_{\Omega }e_{\bar{u}}(x,\tau )\nabla e_{\bar{u}}(x,\tau )\cdot {\bar{f}}_{\theta }^{\text{SDE}}(x,\tau )dx\\ & \;+\frac{1}{2}{\bar{g}}^{2}(\tau )\int_{\Omega }e_{\bar{u}}(x,\tau )(\|\nabla {\bar{u}}_{\theta }(x,\tau ){\|}_{2}^{2}-\|\nabla {\bar{u}}_{\theta }^{\text{SDE}}(x,\tau ){\|}_{2}^{2})dx\\ & \;+\frac{1}{2}{\bar{g}}^{2}(\tau )\int_{\Omega }e_{\bar{u}}(x,\tau )\nabla^{2}(e_{\bar{u}}(x,\tau ))dx+\int_{\Omega }e_{\bar{u}}(x,\tau )\bar{q}(x,\tau )dx. \end{array} \end{array}$$

Note that,

$$\begin{array}{rl} & \frac{1}{2}{\bar{g}}^{2}(\tau )\int_{\Omega }e_{\bar{u}}(x,\tau )(\|\nabla {\bar{u}}_{\theta }(x,\tau ){\|}_{2}^{2}-\|\nabla {\bar{u}}_{\theta }^{\text{SDE}}(x,\tau ){\|}_{2}^{2})dx\\ & =\frac{1}{2}{\bar{g}}^{2}(\tau )\int_{\Omega }e_{\bar{u}}(x,\tau )(\nabla {\bar{u}}_{\theta }(x,\tau )+\nabla {\bar{u}}_{\theta }^{\text{SDE}}(x,\tau ))\cdot \nabla e_{\bar{u}}(x,\tau )dx\\ & =\frac{1}{4}{\bar{g}}^{2}(\tau )\int_{\Omega }(\nabla {\bar{u}}_{\theta }(x,\tau )+\nabla {\bar{u}}_{\theta }^{\text{SDE}}(x,\tau ))\cdot \nabla e_{\bar{u}}^{2}(x,\tau )dx\\ & =-\frac{1}{4}{\bar{g}}^{2}(\tau )\int_{\Omega }(\nabla^{2}{\bar{u}}_{\theta }(x,\tau )+\nabla^{2}{\bar{u}}_{\theta }^{\text{SDE}}(x,\tau ))e_{\bar{u}}^{2}(x,\tau )dx, \end{array}$$

by integration by parts together with the homogeneous boundary conditions of $e_{\bar{u}}$ . Using $\frac{1}{2}\frac{\partial e_{u}^{2}}{\partial \tau }=e_{\bar{u}}\frac{\partial e_{\bar{u}}}{\partial \tau }$ and applying integration by parts again yields

$$\begin{array}{r} \begin{array}{rl} \frac{1}{2}\frac{\partial }{\partial \tau }\|e_{\bar{u}}(\cdot ,\tau ){\|}_{L^{2}(\Omega )}^{2} & =-\frac{1}{2}\int_{\Omega }e_{\bar{u}}^{2}(x,\tau )\nabla \cdot {\bar{f}}_{\theta }^{\text{SDE}}(x,\tau )dx\\ & \;-\frac{1}{4}{\bar{g}}^{2}(\tau )\int_{\Omega }(\nabla^{2}{\bar{u}}_{\theta }(x,\tau )+\nabla^{2}{\bar{u}}_{\theta }^{\text{SDE}}(x,\tau ))e_{\bar{u}}^{2}(x,\tau )dx\\ & \;-\frac{1}{2}{\bar{g}}^{2}(\tau )\int_{\Omega }\|\nabla (e_{\bar{u}}(x,\tau )){\|}_{2}^{2}dx+\int_{\Omega }e_{\bar{u}}(x,\tau )\bar{q}(x,\tau )dx. \end{array} \end{array}$$

From lemma F.1 and ${\bar{u}}_{\theta }\in C^{\infty }(\Omega \times [0,T])$ *,* it follows that there exists L∈ℝ such that $\nabla^{2}{\bar{u}}_{\theta }+\nabla^{2}{\bar{u}}_{\theta }^{\text{SDE}}\geq L$ . Then,

(A 6)
$$\begin{array}{rl} \frac{1}{2}\frac{\partial }{\partial \tau }\|e_{\bar{u}}(\cdot ,\tau ){\|}_{L^{2}(\Omega )}^{2} & \leq \frac{1}{2}\|\nabla \cdot {\bar{f}}_{\theta }^{\;\;SDE}(\cdot ,\tau ){\|}_{L^{\infty }(\Omega )}\|e_{\bar{u}}(\cdot ,\tau ){\|}_{L^{2}(\Omega )}^{2}-\frac{1}{4}{\bar{g}}^{2}(\tau )L\|e_{\bar{u}}(\cdot ,\tau ){\|}_{L^{2}(\Omega )}^{2}\\ & \;-\frac{1}{2}{\bar{g}}^{2}(\tau )\|\nabla (e_{\bar{u}}(\cdot ,\tau )){\|}_{L^{2}(\Omega )}^{2}+\frac{1}{2\epsilon }\|e_{\bar{u}}(\cdot ,\tau ){\|}_{L^{2}(\Omega )}^{2}+\frac{\epsilon }{2}\|\bar{q}(\cdot ,\tau ){\|}_{L^{2}(\Omega )}^{2}, \end{array}$$

which follows from applying Young’s inequality and holds for any ϵ>0 . The Poincaré inequality $K\|e_{\bar{u}}(\cdot ,\tau ){\|}_{L^{2}(\Omega )}\leq \|\nabla e_{\bar{u}}(\cdot ,\tau ){\|}_{L^{2}(\Omega )}$ for some constant K>0 yields

$$\begin{array}{r} \begin{array}{rl} \frac{1}{2}\frac{\partial }{\partial \tau }\|e_{\bar{u}}(\cdot ,\tau ){\|}_{L^{2}(\Omega )}^{2} & \leq \frac{1}{2}\|\nabla \cdot {\bar{f}}_{\theta }^{\text{SDE}}(\cdot ,\tau ){\|}_{L^{\infty }(\Omega )}\|e_{\bar{u}}(\cdot ,\tau ){\|}_{L^{2}(\Omega )}^{2}-\frac{1}{4}{\bar{g}}^{2}(\tau )L\|e_{\bar{u}}(\cdot ,\tau ){\|}_{L^{2}(\Omega )}^{2}\\ & \;-\frac{1}{2}{\bar{g}}^{2}(\tau )K\|e_{\bar{u}}(\cdot ,\tau ){\|}_{L^{2}(\Omega )}^{2}+\frac{1}{2\epsilon }\|e_{\bar{u}}(\cdot ,\tau ){\|}_{L^{2}(\Omega )}^{2}+\frac{\epsilon }{2}\|\bar{q}(\cdot ,\tau ){\|}_{L^{2}(\Omega )}^{2}. \end{array} \end{array}$$

Integration in time yields

$$\begin{array}{r} \begin{array}{rl} \|e_{\bar{u}}(\cdot ,\tau ){\|}_{L^{2}(\Omega )}^{2}\leq & \|e_{\bar{u}}(\cdot ,0){\|}_{L^{2}(\Omega )}^{2}+\epsilon \int_{0}^{\tau }\|\bar{q}(\cdot ,s){\|}_{L^{2}(\Omega )}^{2}ds\\ & +\int_{0}^{\tau }\left(\|\nabla \cdot {\bar{f}}_{\theta }^{\;\;SDE}(\cdot ,s){\|}_{L^{\infty }(\Omega )}-\frac{1}{2}{\bar{g}}^{2}(s)(2K+L)+\frac{1}{\epsilon }\right)\|e_{\bar{u}}(\cdot ,s){\|}_{L^{2}(\Omega )}^{2}ds, \end{array} \end{array}$$

where the term $\|e_{\bar{u}}(\cdot ,0){\|}_{L^{2}(\Omega )}^{2}$ vanishes due to the zero initial condition of $e_{\bar{u}}$ . For ϵ sufficiently small, the bracketed term in the last integral is positive as g is bounded, and hence there exists a positive upper bound for the bracketed term. Therefore, we can apply Gronwall’s inequality to get

(A 7)
$$\begin{array}{r} \begin{array}{rl} & \|e_{\bar{u}}(\cdot ,\tau ){\|}_{L^{2}(\Omega )}^{2}\\ & \leq \epsilon \int_{0}^{\tau }\|\bar{q}(\cdot ,s){\|}_{L^{2}(\Omega )}^{2}\text{d}s\;\text{exp}\left(\int_{0}^{\tau }\left(\|\nabla \cdot {\bar{f}}_{\theta }^{\text{SDE}}(\cdot ,s){\|}_{L^{\infty }(\Omega )}-\frac{1}{2}{\bar{g}}^{2}(s)(2K+L)+\frac{1}{\epsilon }\right)\text{d}s\right). \end{array} \end{array}$$

Using equation (A 5), $\tilde{R}(\theta ,u_{\theta },t)<\delta$ *,* τ<T and the upper bound of the bracketed term, this proves that $\|e_{\bar{u}}(\cdot ,\tau ){\|}_{L^{2}(\Omega )}^{2}<C\delta$ for some constant C>0 independent of δ and τ but dependent on T and ϵ .

Next, we deduce that

(A 8)
$$\begin{array}{r} \int_{0}^{\tau }\|\nabla e_{\bar{u}}(\cdot ,s){\|}_{L^{2}(\Omega )}^{2}\text{d}s<C\delta \end{array}$$

for some C>0 independent of δ . Note that equation (A 6) implies

(A 9)
$$\begin{array}{r} \underset{s\in [0,\tau ]}{\text{min}}\{{\bar{g}}^{2}(s)\}\int_{0}^{\tau }\|\nabla e_{\bar{u}}(\cdot ,s){\|}_{L^{2}(\Omega )}^{2}\text{d}s\leq \epsilon \int_{0}^{\tau }\|\bar{q}(\cdot ,s){\|}_{L^{2}(\Omega )}^{2}\text{d}s-\|e_{\bar{u}}(\cdot ,\tau ){\|}_{L^{2}(\Omega )}^{2}\\ +\int_{0}^{\tau }\left(\|\nabla \cdot {\bar{f}}_{\theta }^{\;\;SDE}(\cdot ,s){\|}_{L^{\infty }(\Omega )}-\frac{1}{2}{\bar{g}}^{2}(s)L+\frac{1}{\epsilon }\right)\|e_{\bar{u}}(\cdot ,s){\|}_{L^{2}(\Omega )}^{2}\text{d}s. \end{array}$$

It follows from equation (A 7) that for any s∈[0,τ] , we have $\|e_{\bar{u}}(\cdot ,s){\|}_{L^{2}(\Omega )}^{2}<C\delta$ . Since g has a positive lower bound by assumptions 2.1 and $\int_{0}^{\tau }\|\bar{q}(\cdot ,s){\|}_{L^{2}(\Omega )}^{2}ds<\delta$ by equation (A 5), this yields equation (A 8) for some C>0 independent of δ .

**Step II:** Finally, we prove that $W_{2}(p_{\theta }^{ODE}(\cdot ,t),p_{\theta }^{\text{SDE}}(\cdot ,t))<C\delta$ . As $e_{\bar{u}}={\bar{u}}_{\theta }-{\bar{u}}_{\theta }^{\text{SDE}}$ in reverse time τ , we have $e_{u}=u_{\theta }-u_{\theta }^{\text{SDE}}$ in forward time t=T−τ with $\nabla e_{u}=\nabla u_{\theta }-\nabla u_{\theta }^{\text{SDE}}$ . Starting from the probability flow ODE of the approximate SDE (2.9) and the approximate drifts $f_{\theta }^{\text{SDE}}$ in (2.6) and $f_{\theta }^{ODE}$ in (2.11), we obtain in forward time

$$\begin{array}{rl} \frac{\text{d}x_{t}}{\text{d}t} & =f_{\theta }^{\text{SDE}}(x_{t},t)+\frac{1}{2}g^{2}(t)\nabla u_{\theta }^{\text{SDE}}(x_{t},t)\\ & =f(x_{t},t)-g^{2}(t)\nabla u_{\theta }(x_{t},t)+\frac{1}{2}g^{2}(t)(\nabla u_{\theta }(x_{t},t)-\nabla e_{u}(x_{t},t))\\ & =f(x_{t},t)-\frac{1}{2}g^{2}(t)\nabla u_{\theta }(x_{t},t)-\frac{1}{2}g^{2}(t)\nabla e_{u}(x_{t},t)\\ & =f_{\theta }^{\;\;ODE}(x_{t},t)-\frac{1}{2}g^{2}(t)\nabla e_{u}(x_{t},t). \end{array}$$

This implies that trajectories traced out by particles obeying equation (2.9) with density $p_{\theta }^{\text{SDE}}$ are close to particles obeying equation (2.10) with density $p_{\theta }^{ODE}$ provided the error $\nabla e_{u}$ is small. The densities $p_{\theta }^{\text{SDE}}$ and $p_{\theta }^{ODE}$ induced by the probability flow ODEs equations (2.9), (2.10) are associated with ${\bar{p}}_{\theta }^{\text{SDE}}$ and ${\bar{p}}_{\theta }^{ODE}$ in reverse time with the corresponding probability flows in reverse time given by

(A 10)
$$\begin{array}{rl} \frac{\text{d}{\bar{x}}_{\tau }}{\text{d}\tau } & =-{\bar{f}}_{\theta }^{\;\;ODE}({\bar{x}}_{\tau },\tau )+\frac{1}{2}{\bar{g}}^{2}(\tau )\nabla e_{\bar{u}}({\bar{x}}_{\tau },\tau ) \end{array}$$

and

(A 11)
$$\begin{array}{rl} \frac{\text{d}{\bar{x}}_{\tau }}{\text{d}\tau } & =-{\bar{f}}_{\theta }^{\;\;ODE}({\bar{x}}_{\tau },\tau ), \end{array}$$

respectively. We equip equations (A 10) and (A 11) with initial conditions ${\bar{x}}_{0}^{\text{SDE}}$ and ${\bar{x}}_{0}^{ODE}$ , respectively, and suppose that ${\bar{x}}_{0}^{\text{SDE}}={\bar{x}}_{0}^{ODE}={\bar{x}}_{0}$ . We denote the solutions to the initial value problems corresponding to equations (A 10) and (A 11) by ${\bar{x}}_{\tau }^{SDE}$ and ${\bar{x}}_{\tau }^{ODE}$ , respectively. For any times 0≤s≤t , let $\phi^{s,t}({\bar{x}}_{s}^{\text{SDE}})$ denote the exact solution of the initial value problem:

$$\begin{array}{r} \left\{ \begin{array}{l} \frac{d{\bar{x}}_{\tau }^{ODE}}{d\tau }=-{\bar{f}}_{\theta }^{\;\;ODE}({\bar{x}}_{\tau }^{ODE},\tau ),\\ {\bar{x}}_{s}^{ODE}={\bar{x}}_{s}^{\text{SDE}}. \end{array}\right. \end{array}$$

Then, the Alekseev–Gröbner formula [[21], theorem 14.5] implies the relation

(A 12)
$$\begin{array}{r} {\bar{x}}_{\tau }^{\text{SDE}}={\bar{x}}_{\tau }^{ODE}+\frac{1}{2}\int_{0}^{\tau }{\bar{g}}^{2}(s)\frac{\partial \phi^{s,t}\left({\bar{x}}_{0}\right)}{\partial {\bar{x}}_{0}}{|}_{{\bar{x}}_{0}={\bar{x}}_{s}^{\text{SDE}}}\nabla e_{\bar{u}}({\bar{x}}_{s}^{\text{SDE}},s)\text{d}s \end{array}$$

between ${\bar{x}}_{\tau }^{\text{SDE}}$ and ${\bar{x}}_{\tau }^{ODE}$ , where $\frac{\partial \phi^{s,t}\left({\bar{x}}_{0}\right)}{\partial {\bar{x}}_{0}}{|}_{{\bar{x}}_{0}={\bar{x}}_{s}^{SDE}}\in {\mathbb{R}}^{d\times d}$ is bounded as ${\bar{f}}_{\theta }^{ODE}\in C^{\infty }({\mathbb{R}}^{d}\times [0,T];{\mathbb{R}}^{d})$ by the definition of $f_{\theta }^{ODE}$ in equation (2.11). Now let $\Gamma_{\tau }$ denote the set of couplings of ${\bar{p}}_{\theta }^{SDE}(\cdot ,\tau )$ and ${\bar{p}}_{\theta }^{ODE}(\cdot ,\tau )$ . Let ${\bar{p}}_{\tau }({\bar{x}}_{\tau }^{\text{SDE}},{\bar{x}}_{\tau }^{ODE})$ be the joint distribution of $\left({\bar{x}}_{\tau }^{\text{SDE}},{\bar{x}}_{\tau }^{ODE}\right)$ defined by the pushforward $T_{\#}\pi$ of π , where $T:{\bar{x}}_{0}\mapsto ({\bar{x}}_{\tau }^{\text{SDE}},{\bar{x}}_{\tau }^{ODE})$ is defined by evolving equations (A 10) and (A 11) with initial condition ${\bar{x}}_{0}$ . Clearly, ${\bar{p}}_{\tau }$ is a coupling, so ${\bar{p}}_{\tau }\in \Gamma_{\tau }$ . This yields the bound

$$\begin{array}{rl} W_{2}^{2}({\bar{x}}_{\tau }^{\text{SDE}},{\bar{x}}_{\tau }^{ODE}) & =\underset{\gamma \in \Gamma_{\tau }}{\inf}\iint_{\Omega \times \Omega }\|{\bar{x}}_{\tau }^{SDE}-{\bar{x}}_{\tau }^{ODE}{\|}_{2}^{2}\gamma ({\bar{x}}_{\tau }^{\text{SDE}},{\bar{x}}_{\tau }^{ODE})d{\bar{x}}_{\tau }^{ODE}\bar{d}x_{\tau }^{\text{SDE}}\\ & \leq \iint_{\Omega \times \Omega }\|{\bar{x}}_{\tau }^{\text{ODE}}-{\bar{x}}_{\tau }^{\text{SDE}}{\|}_{2}^{2}{\bar{p}}_{\tau }({\bar{x}}_{\tau }^{\text{ODE}},{\bar{x}}_{\tau }^{\text{SDE}})d{\bar{x}}_{\tau }^{\text{SDE}}d{\bar{x}}_{\tau }^{\text{ODE}}\\ & =\iint_{\Omega \times \Omega }\|{\bar{x}}_{\tau }^{\text{ODE}}-{\bar{x}}_{\tau }^{\text{SDE}}{\|}_{2}^{2}{\bar{p}}_{\tau }({\bar{x}}_{\tau }^{\text{ODE}}|{\bar{x}}_{\tau }^{\text{SDE}})d{\bar{x}}_{\tau }^{\text{ODE}}{\bar{p}}_{\tau }^{SDE}({\bar{x}}_{\tau }^{\text{SDE}})d{\bar{x}}_{\tau }^{\text{SDE}}, \end{array}$$

where ${\bar{p}}_{\tau }^{\text{SDE}}$ denotes the distribution of ${\bar{x}}_{\tau }^{\text{SDE}}$ . Due to the bounds of f,g in assumption 2.1, ${\bar{x}}_{\tau }^{\text{SDE}}$ is bounded in $L^{\infty }(\Omega \times [0,T])$ . Further, we have a deterministic relation between ${\bar{x}}_{\tau }^{\text{ODE}}$ and ${\bar{x}}_{\tau }^{\text{SDE}}$ given by equation (A 12), therefore

$$\begin{array}{r} p({\bar{x}}_{\tau }^{\text{ODE}}|{\bar{x}}_{\tau }^{\text{SDE}})=\delta \left({\bar{x}}_{\tau }^{\text{ODE}}-\left({\bar{x}}_{\tau }^{\text{SDE}}-\frac{1}{2}\int_{0}^{\tau }{\bar{g}}^{2}(s)\frac{\partial \phi^{s,t}\left({\bar{x}}_{0}\right)}{\partial {\bar{x}}_{0}}{|}_{{\bar{x}}_{0}={\bar{x}}_{s}^{\text{SDE}}}\nabla e_{\bar{u}}({\bar{x}}_{s}^{\text{SDE}},s)ds\right)\right). \end{array}$$

Integrating over ${\bar{x}}_{\tau }^{\text{ODE}}$ gives us

$$\begin{array}{rl} & W_{2}^{2}({\bar{x}}_{\tau }^{\text{SDE}},{\bar{x}}_{\tau }^{ODE})\\ & \leq \int_{\Omega }{\left\|\frac{1}{2}\int_{0}^{\tau }{\bar{g}}^{2}(s)\frac{\partial \phi^{s,t}\left({\bar{x}}_{0}\right)}{\partial {\bar{x}}_{0}}{|}_{{\bar{x}}_{0}={\bar{x}}_{s}^{SDE}}\nabla e_{\bar{u}}({\bar{x}}_{s}^{\text{SDE}},s)ds\right\|}_{2}^{2}{\bar{p}}_{\tau }({\bar{x}}_{\tau }^{\text{SDE}})d{\bar{x}}_{\tau }^{\text{SDE}}\\ & \leq \frac{1}{2}\int_{0}^{\tau }\int_{\Omega }{\left\|{\bar{g}}^{2}(s)\frac{\partial \phi^{s,t}\left({\bar{x}}_{0}\right)}{\partial {\bar{x}}_{0}}{|}_{{\bar{x}}_{0}={\bar{x}}_{s}^{\text{SDE}}}\nabla e_{\bar{u}}({\bar{x}}_{s}^{\text{SDE}},s)\right\|}_{2}^{2}{\bar{p}}_{\tau }({\bar{x}}_{\tau }^{\text{SDE}})d{\bar{x}}_{\tau }^{\text{SDE}}\;ds\\ & \leq \frac{1}{2}\|{\bar{p}}_{\tau }^{\text{SDE}}(\cdot ){\|}_{L^{\infty }(\Omega \times [0,T])}\int_{0}^{\tau }{\left\|{\bar{g}}^{2}(s)\frac{\partial \phi^{s,t}\left({\bar{x}}_{0}\right)}{\partial {\bar{x}}_{0}}{|}_{{\bar{x}}_{0}=\;\cdot }\nabla e_{\bar{u}}(\cdot ,s)\right\|}_{L^{2}(\Omega )}^{2}ds\\ & \leq \frac{1}{2}\|{\bar{p}}_{\tau }^{\text{SDE}}(\cdot ){\|}_{L^{\infty }(\Omega \times [0,T])}\|{\bar{g}}^{2}(\cdot ){\|}_{L^{\infty }([0,T])}\\ & \;\;\;{\left\|\frac{\partial \phi^{s,t}\left({\bar{x}}_{0}\right)}{\partial {\bar{x}}_{0}}{|}_{{\bar{x}}_{0}=\;\cdot }\right\|}_{L^{\infty }(\Omega \times [0,T{]}^{2})}\int_{0}^{\tau }\|\nabla e_{\bar{u}}(\cdot ,s){\|}_{L^{2}(\Omega )}^{2}ds. \end{array}$$

Note that g is bounded by assumption 2.1, the Jacobian $\frac{\partial \phi^{s,t}\left({\bar{x}}_{0}\right)}{\partial {\bar{x}}_{0}}$ is bounded and equation (A 8) holds for some C>0 independent of δ . Thus, we obtain $W_{2}^{2}({\bar{x}}_{\tau }^{SDE},{\bar{x}}_{\tau }^{ODE})<C\delta$ for some C>0 independent of δ , and hence we have established the required bound on Wasserstein distance.∎

## Appendix B. Derivation of the ODE–SDE gap for the potential model associated with the approximate Fokker–Planck equation

In this section, we derive the ODE–SDE gap for the potential model associated with the approximate Fokker–Planck equation, given by corollary B.1.

**Corollary B.1.** *Assume that assumptions* 2.1 *and* 3.1 *hold. Further, assume that the neural network* $\phi_{\theta }\in C^{\infty }(\Omega \times [0,T])$ *in* (3.1) *is determined such that* $\phi_{\theta }$ *obeys the terminal condition* $\phi_{T}$ *and Dirichlet boundary conditions* $\phi_{B}^{SDE}$ *on* ∂Ω×[0,T] *, and* $\tilde{R}(\theta ,\phi_{\theta },t)$ *in* (2.19) *satisfies* $\tilde{R}(\theta ,\phi_{\theta },t)<\delta$ . *Then,* $W_{2}(p_{\theta }^{ODE}(\cdot ,t),p_{\theta }^{\text{SDE}}(\cdot ,t))<C\delta$ *for some* C>0 *independent of* δ .

*Proof*. By the definition of $e_{u}$ in the proof of theorem 3.1, we have

$$\begin{array}{r} u_{\theta }(x,t)=u_{\theta }^{\text{SDE}}(x,t)+e_{u}(x,t), \end{array}$$

thus implying

$$\begin{array}{rl} \phi_{\theta }(x,t) & =u_{\theta }^{\text{SDE}}(x,t)+e_{u}(x,t)+\log Z_{t}\\ & =u_{\theta }^{\text{SDE}}(x,t)+\log Z_{T}+(e_{u}(x,t)-\log Z_{T}+\log Z_{t})\\ & =u_{\theta }^{\text{SDE}}(x,t)+\log Z_{T}+{\tilde{e}}_{u}(x,t), \end{array}$$

where we have set

$$\begin{array}{rl} {\tilde{e}}_{u}(x,t) & =e_{u}(x,t)-\log Z_{T}+\log Z_{t}\\ & =\phi_{\theta }(x,t)-u_{\theta }^{SDE}(x,t)-\log Z_{T} \end{array}$$

as the error between $\phi_{\theta }$ and $u_{\theta }^{\text{SDE}}+\log Z_{T}$ . Since $u_{\theta }^{\text{SDE}}$ is a solution of equation (2.17), it follows that $u_{\theta }^{\text{SDE}}+\log Z_{T}$ is also a solution of equation (2.17). Following similar arguments as in equation (A 9), we deduce that

$$\begin{array}{rl} \int_{t}^{T}\|\nabla u_{\theta }^{\text{SDE}}(\cdot ,s)-\nabla \phi_{\theta }(\cdot ,s){\|}_{L^{2}(\Omega )}^{2}ds & =\int_{t}^{T}\|\nabla u_{\theta }^{\text{SDE}}(\cdot ,s)+\nabla \log Z_{T}-\nabla \phi_{\theta }(\cdot ,s){\|}_{L^{2}(\Omega )}^{2}ds\\ & =\int_{t}^{T}\|\nabla {\tilde{e}}_{u}(\cdot ,s){\|}_{L^{2}(\Omega )}^{2}ds\\ & <C\delta \end{array}$$

for some C>0 independent of δ . Thus, $W_{2}(p_{\theta }^{ODE}(\cdot ,t),p_{\theta }^{\text{SDE}}(\cdot ,t))<C\delta$ follows by applying the steps in the proof of theorem 3.1 with $\phi_{\theta }$ substituted in place of $u_{\theta }$ .∎

## Appendix C. Further numerical evidence

In this section, we provide additional numerical results. This includes figures 3 and 4, which show how the Fokker–Planck residual is correlated with $W_{2}(p_{\theta }^{ODE},p_{\theta }^{\text{SDE}})$ and the score-matching objective in our experiments, as well as tables 1–3, which explicitly list calculated $W_{2}$ distances between various distributions at different regularization strengths.

**Table 1.** Estimated values of $W_{2}^{2}(p_{\theta }^{ODE}(\cdot ,0),p_{\theta }^{\text{SDE}}(\cdot ,0))$ for each distribution and $w_{R}$ value.

$w_{R}$	mixture	circles	checkerboard
0.0	0.011599	0.013401	0.027196
0.1	0.003107	0.005283	0.007160
1	0.002901	0.003090	0.003528
10	0.002208	0.001704	0.002104

**Table 2.** Estimated values of $W_{2}^{2}(p_{\theta }^{ODE}(\cdot ,0),p_{0})$ for each distribution and differing $w_{R}$ values.

$w_{R}$	mixture	circles	checkerboard
0.0	0.010967	0.014354	0.024416
0.1	0.006039	0.007373	0.011386
1	0.005280	0.008280	0.011428
10	0.017225	0.009378	0.033052

**Table 3.** Estimated values of $W_{2}^{2}(p_{\theta }^{\text{SDE}}(\cdot ,0),p_{0})$ for each distribution and differing $w_{R}$ values.

$w_{R}$	mixture	circles	checkerboard
0.0	0.002180	0.002268	0.003681
0.1	0.002940	0.003323	0.005387
1	0.004271	0.004541	0.011552
10	0.015989	0.009097	0.032158

In figure 3, we have plotted the results from each of our experiments with $w_{R}\in \{0.0,0.1,1.0,\;10.0\}$ *and* the three two-dimensional distributions (Gaussian mixture, concentric circles and checkerboard) on to a graph that shows the resulting Fokker–Planck residuals and Wasserstein distances between ODE- and SDE-based samples. We use three million samples from each distribution to numerically estimate the Wasserstein distances, and the Fokker–Planck residual is calculated using quadrature over a fine discretization. We observe a clear trend showing that a smaller Fokker–Planck residual is associated with a smaller ODE-SDE gap, which is consistent with the theoretical analysis.

Figure 4 shows that the reduction of the Fokker–Planck residual using our regularization technique generally led to an increase in the resulting score-matching loss. This effect is most notable for the highest regularization level $w_{R}=10$ (corresponding to the three points grouped in the bottom right of this figure), and indicates that this is over-regularizing. This is not surprising, as with a high regularization weighting the optimizer can be expected to de-prioritize the score-matching loss in favour of finding a score for which the corresponding Fokker–Planck equation can be solved more accurately.

The remainder of this section contains tables 1–3, which list Wasserstein distances resulting from our experiments for each pairing of $p_{\theta }^{ODE},p_{\theta }^{\text{SDE}},p_{0}$ . We summarize these results together here. Table 1 list the Wasserstein distances between $p_{\theta }^{ODE}$ and $p_{\theta }^{\text{SDE}}$ , similarly to figure 3, and demonstrates that, as expected, the ODE–SDE gap decreases as the regularization strength $w_{R}$ increases. Table 2 list the distances between $p_{\theta }^{ODE}$ and $p_{0}$ . In all experiments, we observe a reduction in this distance for $w_{R}\in \left\{0.1,1\right\}$ when compared with the unregularized case, thus we can conclude that for moderate regularization an improvement in ODE samples can be obtained. At the higher $w_{R}=10$ level, the distance grows considerably, indicating over-regularization. Finally, table 3 list the distances between $p_{\theta }^{\text{SDE}}$ and $p_{0}$ . This table reveals monotonic degredation of SDE-based sample quality and we conclude that the Fokker–Planck-based regularization is detrimental to the sampling distribution obtained by the approximate reverse SDE. As a result, this type of technique will have the most benefit in applications where ODE-based methods are required.

## Appendix D. Non-zero curl of conventional score-parametrization

In this section, we show figure 5 illustrating that the conventional score-parameterization often results in score approximations with a non-zero curl. This demonstrates that the conventional score approximation is not a conservative vector field. However, this is a desirable property as the ground truth is a gradient field and motivates the potential parametrization considered in this article.

## Appendix E. Likelihood weighting function

The likelihood weighting function $\lambda (t)=g^{2}(t)$ ensures that equation (2.5) together with the Kullback–Leibler divergence $D_{KL}$ from the true terminal distribution p(⋅,T) to the given prior distribution π yields an upper bound on the Kullback–Leibler divergence from the target distribution p(⋅,0) to the model distribution $p_{\theta }^{\text{SDE}}(\cdot ,0)$ . Here, $p_{\theta }^{\text{SDE}}(\cdot ,0)$ refers to the distribution of samples obtained by simulating a neural approximation of particle dynamics equation (2.2), which we introduce in equation (2.7). More precisely, it holds that

$$\begin{array}{rl} D_{KL}(p(\cdot ,0)∥p_{\theta }^{\text{SDE}}(\cdot ,0)) & \leq D_{KL}(p(\cdot ,T)∥\pi (\cdot ))+\frac{T}{2}L_{SM}(\theta ,s_{\theta },g^{2}). \end{array}$$

Other weighting functions also yielded very good results [22] for particular choices of forward SDEs. However, there are no theoretical guarantees that alternative weightings would yield good results for arbitrary choices of forward SDEs.

Similarly to the model distribution $p_{\theta }^{\text{SDE}}(\cdot ,0)$ of the neural approximation of the particle dynamics equation 2.2, one can also consider the distribution of samples $p_{\theta }^{ODE}(\cdot ,0)$ obtained by simulating the neural approximation of particle dynamics equation (2.3) and we will formally introduce $p_{\theta }^{ODE}(\cdot ,0)$ in §2d. A bound of the Kullback–Leibler divergence from the target distribution p(⋅,0) to the model distribution $p_{\theta }^{ODE}(\cdot ,0)$ has been derived in [14] and is given by

$$\begin{array}{r} D_{KL}(p(\cdot ,0)∥p_{\theta }^{ODE}(\cdot ,0))=D_{KL}(p(\cdot ,T)∥\pi (\cdot ))+\frac{T}{2}L_{SM}(\theta ,s_{\theta },g^{2})+\frac{T}{2}L_{Diff}(\theta ,s_{\theta },g^{2}), \end{array}$$

where

$$\begin{array}{r} L_{Diff}(\theta ,s_{\theta },\lambda ):=E_{\begin{array}{c} t∼U(0,T)\\ x_{t}∼p(x_{t},t) \end{array}}\left[\lambda (t){\left(\nabla \log p(x,t)-s_{\theta }(x,t)\right)}^{T}\left(\nabla \log p_{\theta }^{ODE}(x,t)-s_{\theta }(x,t)\right)\right]dt. \end{array}$$

Upper bounds for $L_{Diff}(\theta ,s_{\theta },g^{2})$ have been derived in [12,14], and training schemes based on minimizing these upper bounds have been proposed.

## Appendix F. Lower bound on $\nabla^{2}u_{\theta }^{\text{SDE}}$

The proof of theorem 3.1 requires a preliminary result on the existence of a lower bound of $\nabla^{2}u_{\theta }^{\text{SDE}}$ , given by the following lemma.

**Lemma F.1.** *Let* t∈[0,T] *, let* $\Omega \subset {\mathbb{R}}^{d}$ *be a bounded domain with* $\partial \Omega \in C^{\infty }$ *, and assume that assumptions* 2.1 *hold. Assume that* $p_{\theta }^{\text{SDE}}$ *satisfies* equation (2.15) *on* Ω *with terminal condition* π *restricted to* Ω *and Dirichlet boundary conditions* $p_{B}^{\text{SDE}}$ *on* ∂Ω×[0,T] *, with* $u_{\theta }^{\text{SDE}}=\log p_{\theta }^{\text{SDE}}$ . *Then there exists* C∈ℝ *such that* $\nabla^{2}{\bar{u}}_{\theta }^{\text{SDE}}\geq C$ *on* Ω×[0,T] *for* ${\bar{u}}_{\theta }^{\text{SDE}}(\cdot ,\tau )=u_{\theta }^{\text{SDE}}(\cdot ,T-\tau )$ *for* τ∈[0,T] .

*Proof*. By construction $u_{\theta }^{\text{SDE}}=\log p_{\theta }^{\text{SDE}}$ where $p_{\theta }^{\text{SDE}}$ solves equation (2.15) with terminal condition $p_{\theta }^{\text{SDE}}(x,T)=\pi$ and Dirichlet boundary data $p_{B}^{\text{SDE}}$ . Using the reverse time variable τ=T−t , we can write the reverse dynamics for ${\bar{p}}_{\theta }^{\text{SDE}}(x,\tau )$ as

$$\begin{array}{r} \frac{\partial {\bar{p}}_{\theta }^{\text{SDE}}}{\partial \tau }(x,\tau )=\nabla \cdot ({\bar{f}}_{\theta }^{\text{SDE}}(x,\tau ){\bar{p}}_{\theta }^{\text{SDE}}(x,\tau ))+\frac{1}{2}{\bar{g}}^{2}(\tau )\nabla^{2}({\bar{p}}_{\theta }^{\text{SDE}}(x,\tau )), \end{array}$$

with initial data ${\bar{p}}_{\theta }^{\text{SDE}}(\cdot ,0)=\pi$ and Dirichlet boundary conditions ${\bar{p}}_{\theta }^{\text{SDE}}(x,\tau )=p_{B}^{\text{SDE}}(x,T-\tau )$ for (x,τ)∈∂Ω×[0,T] . Under assumptions 2.1 on $f,g,u_{\theta }$ *,* we have that ${\bar{p}}_{\theta }^{SDE}\in C^{\infty }(\Omega \times [0,T])$ by [[23], Thm. 8.3.4]. Furthermore, ${\bar{p}}_{\theta }^{\text{SDE}}$ is strictly positive on Ω . Since ${\bar{p}}_{\theta }^{\text{SDE}}\in C^{\infty }(\Omega \times [0,T])$ there exist $k_{1},k_{2}>0$ such that $\|\nabla {\bar{p}}_{\theta }^{\text{SDE}}(x,\tau ){\|}_{2}<k_{1}$ and $|\nabla^{2}{\bar{p}}_{\theta }^{\text{SDE}}(x,\tau )|<k_{2}$ for all (x,τ)∈Ω×[0,T] . As ${\bar{p}}_{\theta }^{\text{SDE}}$ is strictly positive, there exists $k_{0}>0$ such that ${\bar{p}}_{\theta }^{\text{SDE}}(x,\tau )>k_{0}$ on Ω×[0,T] . Computing directly from ${\bar{u}}_{\theta }^{\text{SDE}}=\log{\bar{p}}_{\theta }^{\text{SDE}}$ we obtain that

$$\begin{array}{rl} \nabla^{2}{\bar{u}}_{\theta }^{\text{SDE}}(x,\tau ) & =\frac{{\bar{p}}_{\theta }^{\text{SDE}}(x,\tau )\nabla^{2}{\bar{p}}_{\theta }^{\text{SDE}}(x,\tau )-\|\nabla {\bar{p}}_{\theta }^{\text{SDE}}(x,\tau ){\|}_{2}^{2}}{{\bar{p}}_{\theta }^{\text{SDE}}(x,\tau {)}^{2}}\\ & =\frac{\nabla^{2}{\bar{p}}_{\theta }^{\text{SDE}}(x,\tau )}{{\bar{p}}_{\theta }^{\text{SDE}}(x,\tau )}-{\left\|\frac{\nabla {\bar{p}}_{\theta }^{\text{SDE}}(x,\tau )}{{\bar{p}}_{\theta }^{\text{SDE}}(x,\tau )}\right\|}_{2}^{2}. \end{array}$$

This results in the bound

$$\begin{array}{r} |\nabla^{2}{\bar{u}}_{\theta }^{\text{SDE}}(x,\tau )|\leq \left|\frac{\nabla^{2}{\bar{p}}_{\theta }^{\text{SDE}}(x,\tau )}{{\bar{p}}_{\theta }^{\text{SDE}}(x,\tau )}\right|+{\left\|\frac{\nabla {\bar{p}}_{\theta }^{\text{SDE}}(x,\tau )}{{\bar{p}}_{\theta }^{\text{SDE}}(x,\tau )}\right\|}_{2}^{2}\leq \left|\frac{k_{2}}{k_{0}}\right|+{\left(\frac{k_{1}}{k_{0}}\right)}^{2}, \end{array}$$

which yields the required bound.∎
